# A Medicinal Halophyte *Ipomoea pes-caprae* (Linn.) R. Br.: A Review of Its Botany, Traditional Uses, Phytochemistry, and Bioactivity

**DOI:** 10.3390/md20050329

**Published:** 2022-05-17

**Authors:** Ganiyu Akinniyi, Jeonghee Lee, Hiyoung Kim, Joon-Goo Lee, Inho Yang

**Affiliations:** 1 Department of Convergence Study on the Ocean Science and Technology, Korea Maritime and Ocean University, Busan 49112, Korea; gaakinniyi@g.kmou.ac.kr (G.A.); jeonghee2551@g.kmou.ac.kr (J.L.); 2 Department of Biomedical Science and Engineering, Konkuk University, Seoul 05029, Korea; reihyoung@konkuk.ac.kr; 3 Department of Food Biotechnology, Dong-A University, Busan 49315, Korea

**Keywords:** *Ipomoea pes-caprae*, halophyte natural products, traditional medicine, phytochemistry, halophyte

## Abstract

*Ipomoea pes-caprae* (Linn.) R. Br. (Convolvulaceae) is a halophytic plant that favorably grows in tropical and subtropical countries in Asia, America, Africa, and Australia. Even though this plant is considered a pan-tropical plant, *I. pes-caprae* has been found to occur in inland habitats and coasts of wider areas, such as Spain, Anguilla, South Africa, and Marshall Island, either through a purposeful introduction, accidentally by dispersal, or by spreading due to climate change. The plant parts are used in traditional medicine for treating a wide range of diseases, such as inflammation, gastrointestinal disorders, pain, and hypertension. Previous phytochemical analyses of the plant have revealed pharmacologically active components, such as alkaloids, glycosides, steroids, terpenoids, and flavonoids. These phytoconstituents are responsible for the wide range of biological activities possessed by *I. pes-caprae* plant parts and extracts. This review arranges the previous reports on the botany, distribution, traditional uses, chemical constituents, and biological activities of *I. pes-caprae* to facilitate further studies that would lead to the discovery of novel bioactive natural products from this halophyte.

## 1. Introduction

**Figure 1:**
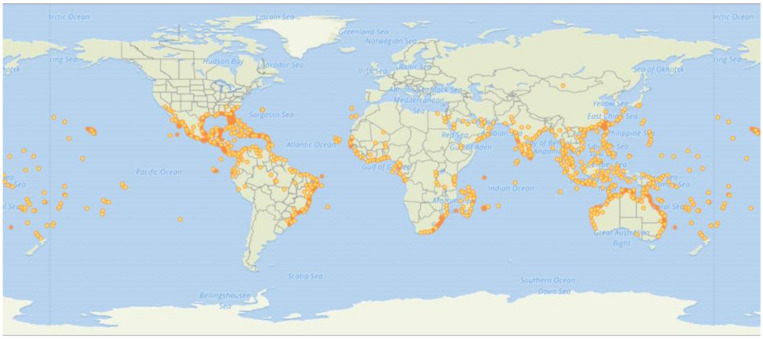
A map showing the global distribution of *I. pes-caprae*.

*Ipomoea pes-caprae* (Linn.) R. Br. is a widely distributed halophytic plant belonging to the family Convolvulaceae. This species trails and colonizes sand dunes along tropical and subtropical coastal beaches, preventing the erosion of dunes [1]. Moreover, *I. pes-caprae* occurs across coastal strands of tropical North and South America, Asia, East- and West-Central Africa, and Australia (Figure 1) [2]. This plant is commonly called beach morning glory, bay hops, or railroad vine.

Traditionally, *I. pes-caprae* has been used for many medicinal purposes. For example, Australian Aborigines apply the heated leaves to wounds, skin infections, and inflamed sores, as well as to stings from poisonous fishes, manta rays, and insects [3]. In some parts of India, it is used in ritual baths to alleviate evil spirits. In Brazil, it serves an important role in traditional medicine to treat inflammation, gastrointestinal disorders, and pain [4]. Infusions of *I. pes-caprae* leaves have been recommended for treating hypertension and kidney ailments. At the same time, decoctions of the same plant are used to treat digestive disorders, internal and external pains, dysentery, inflammations, fatigue, strain, arthritis, and rheumatism [3].

Many natural product chemistry studies have been conducted on various parts of *I. pes-caprae*, which provides a scientific basis for certain uses of the plant in folk medicine. For instance, Pothula and Kanikaram evaluated the in vitro antimalarial activity of the root extracts of *I. pes-caprae*, which indicated that the methanolic extract possesses an excellent antimalarial activity with an half-maximal inhibitory concentration (IC_50_) value of 15 µg/mL against *Plasmodium falciparum* strain 3D7 [5]. The phytochemical screening of the extracts of *I. pes-caprae* also revealed the presence of secondary metabolites, such as alkaloids, triterpenes, flavonoids, tannins, coumarins, carbohydrates, phenols, saponins, phlobatannins, and steroids [5]. The known uses of this plant in traditional medicine can be attributed to these secondary metabolites.

In general, secondary metabolites with various chemical scaffolds have been isolated from *I. pes-caprae*, exhibiting a wide range of biological activities. This review classifies and categorizes these isolated natural products from *I. pes-caprae* according to their biological activities from previous studies.

## 2. Botany

The root of *I. pes-caprae* is large and thick, about 3 m long, and 5 cm in diameter. The stem of this plant is succulent, running along the ground and rooting at the nodes [6]. The stem is green in color, herbaceous, prostrate, cylindrical, up to 30 cm height bearing numerous branches, and 0.3–0.4 cm in thickness; the odor and taste are characteristic with a smooth outer surface [7]. The leaves are simple, alternately arranged, dark green, and glabrous. The leaf shape can be variable but is typically ovate, orbicular, or oblong. The leaf base is truncate to shallowly cordate, and the apex is usually notched to deeply cleft but is sometimes rounded or truncated. The petioles vary in length, ranging from 2 to 15 cm. On young leaves, the petioles are commonly reddish, becoming yellowish-green as they age. There is a pair of nectar-producing glands on the underside of each leaf blade at its juncture with the petiole. These nectaries are red on new leaves, turning black with age, attracting ants, which defend the plant against herbivorous insects [6]. Its characteristic goat footprint-like leaf shape is a descriptive anatomical feature for naming the species. *I. pes-caprae* has characteristic pinkish lavender funnel-shaped flowers (6.4–7.6 cm wide) that bloom throughout the summer and fall [3]. The fruits are ovoid to flattened-globose, and capsules are dehiscent, usually measuring 1.3–1.9 cm long and wide. The seed is rounded to trigonous (three-sided), covered with dense, velvety hairs, and is 1.5–2.5 cm long [6].

*I. pes-caprae* found distributed on sandy beaches or in sunny roadside areas with a characteristic high-temperature and dry environment exhibits great salt tolerance and drought resistance [8]. As a typical halophyte, *I. pes-caprae* has a high-nutrient-utilization efficiency and plays important roles in sand fixation, wind resistance, landscape greening, and ecological restoration of the vegetation in tropical and subtropical coral islands and coastal zones [9]. The development of adventitious roots are important for the growth and survival of *I. pes-caprae*. When the stems are broken by a storm or when the stolons closer to the sea are washed away, they regrow quickly by vegetative reproduction [10]. The vines of *I. pes-caprae* easily sprout adventitious roots, and the depth of primary roots can reach 3 m, to make sure the plant is able to access water [11]. As a measure of reducing or eliminating osmotic stress and the toxic ions from seawater, *I. pes-caprae* accumulates large amounts of saline ions in vivo and has evolved a series of salt-tolerant mechanisms as an adaptive response to high-salt environments [12]. Additionally, the waxy and succulent leaf surface helps to prevent osmotic-stress damage. This characteristic allows *I. pes-caprae* to absorb water with ease and limit the stomata transpiration, and thus aid strong drought tolerance under high temperature and strong light [12]. *I. pes-caprae* is more tolerant to saline stress than to water stress, because its ability to accumulate solutes decreases at high-water-stress levels. Thus, it has a low inherent water-stress tolerance for long drought periods [13].

## 3. Establishment of *I. pes-caprae* in New Habitats

According to the Global Register of Introduced and Invasive Species, a few *I. pes-caprae* occur in Spain, Anguilla, and South Africa, as invasive species [14]. Similarly, there are reports of this species on Marshall Island, where it is referred to as ‘topo’ and considered an invasive species. Even though the plant is unknown to many in Marshall Island, the stems of this spreading vine are sometimes used for children’s jump ropes and medicinal use, although not clearly defined.

Interestingly, there are a few reports on the occurrence of large populations of *I. pes-caprae* in inland habitat, a plant originally known for colonizing sand dunes along coastal beaches [15,16]. Ridley stated that *I. pes-caprae* was never found inland, except in temporal cases where the seeds were dispersed in sand [17]. Contrary to this, in 2005, Devall and Thien revealed a wide population of *I. pes-caprae* growing inland on Lake Nicaragua’s shores, believed to have been introduced inadvertently (conveyed by traveling ships to Granada), or purposefully due to its medicinal purpose [15]. However, the landward extent of the species is often limited by dispersal, competition, and shading by plants beyond the strand [1].

## 4. Traditional Uses

According to the literature, *Ipomoea pes-caprae* is widely used in folk medicine to treat several diseases. The use of this plant in traditional medicine has been documented in Brazil, Mexico, Thailand, Indonesia, Bahamas, Nigeria, Papua New Guinea, French Guiana, and India [3,18,19,20,21,22,23]. Different parts of the plant, such as the leaves, roots, seeds, and stem sap, have been employed traditionally; however, the most used part of *I. pes-caprae* is the leaves – either the dried leaves or the fresh ones. For instance, the dried leaves of the plant are used to treat arthritis in Nigeria [21], while the young leaves are boiled in coconut oil to treat sores in Indonesia. There are similarities regarding the traditional use of this plant to treat diseases/disorders from one country to another.

For treating gastrointestinal-related disorders and symptoms, such as dysentery, ulcer, abdominal pain, and cramps, people from Mexico and Indonesia enhance the leaves of *I. pes-caprae* as infusion or decoction [24]. The roots are employed for their diuretic and mild laxative actions in French Guiana [1]. In Papua New Guinea, the leaves are chewed to relieve stomach aches while other plant parts, such as the seeds, are chewed with areca nut to soothe abdominal pains and cramps among the Thais [19]. In Mexico City, *I. pes-caprae* is available as a drug ‘rinonina’ in numerous herbal markets and healthfood stores where the infusions and decoctions are used to treat functional digestive disorders [3].

*I. pes-caprae* leaves are used in Thailand, Malaysia, China, Mauritius, and Australia for treating skin and joint diseases with associated pain and inflammations, such as dermatitis, boil, bedsores, and stings from jellyfish and stony fish, arthritis, and rheumatism [20,22,23]. Topical application of *I. pes-caprae* is used in this regard. Similar to the method used in Malaysia where the juice is squeezed from the leaves and applied to the area of the fish stings, the leaves are grounded and made into a paste form with distilled vinegar in Thailand. The liquid is eventually squeezed out and applied to the affected area [25]. The topical application of *I. pes-caprae* leaves is similar in Australia and Papua New Guinea, where the leaves are heated on fire and applied to the sores and stings [3]. Other plant parts, such as the stem sap, have also been used for treating sore eyelids, boils, and earaches.

To alleviate strain, fatigue, and physical weakness, decoctions of the plant are used as herbal baths [26] while an infusion of the leaves in hot water is taken orally in Bimini for the same purpose [1].

Other uses of *I. pes-caprae* have been reported. For example, the infusion of the leaves is used for treating hypertension and kidney ailments [3]. *Ipomoea pes-caprae* as “Vriddhadaru” in Ayurvedic medicine has been reported to be effective in the management of diabetes [27]. In Mauritius, people suffering from hemorrhoids either take a bath with a decoction of the plant or sit in a recipe containing a hot decoction so that the vapor reaches the hemorrhoids [20]. As a ritual bath, it is believed that the use of *Ipomoea pes-caprae* wards off evil spirits [28].

As a comment, the traditional use of *I. pes-caprae* to treat strain, fatigue, weakness, pain, and inflammation resulting from sting, arthritis, and rheumatism; intestinal disorders, such as ulcers, dysentery, and cramps; skin diseases, such as boils, bedsores, and dermatitis; and diabetes have been well documented in the literature. Meanwhile, the evident methods of herbal preparation used across these regions include infusion, decoction, herbal poultices, and herbal oils. In addition, these herbal forms are mostly administered either orally or topically.

## 5. Phytochemistry of *I. pes-caprae*

The major components of *I. pes-caprae* are alkaloids, norisoprenoids, phenols, terpenoids, steroids, and glycosides. According to the available literature, approximately 93 major compounds have been identified from *I. pes-caprae*, including one nortropane alkaloid (**1**); two norisoprenoids (**2** and **3**); twenty-three phenols (**4**–**26**); twenty-seven terpenoids (**27**–**53**); two steroids (**54** and **55**); thirty-six glycosides (**56**–**91**); and other compounds, such as xanthoxyline (2-hydroxy-4,6-dimethoxyacetophenone) (**92**) and 2,4-dihydroxy-6-methoxyacetophenone (**93**).

### 5.1. Alkaloid

**Figure 2:**
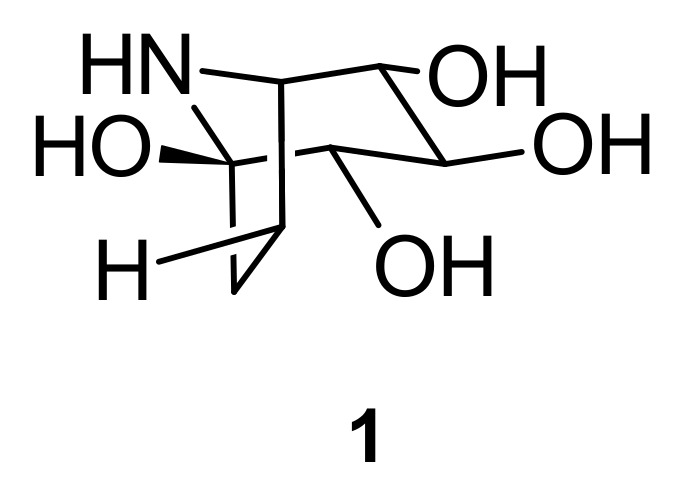
The alkaloid (**1**) from *I. pes-caprae*.

Calystegine B2 (**1**) (Figure 2) has been isolated from *I. pes-caprae*. This alkaloid is a member of nortropane alkaloid. Compound **1** is a tetrahydroxy congener that binds specifically to the active sites of glycosidases, inhibiting the enzymes [29,30].

### 5.2. Norisoprenoids

**Figure 3:**
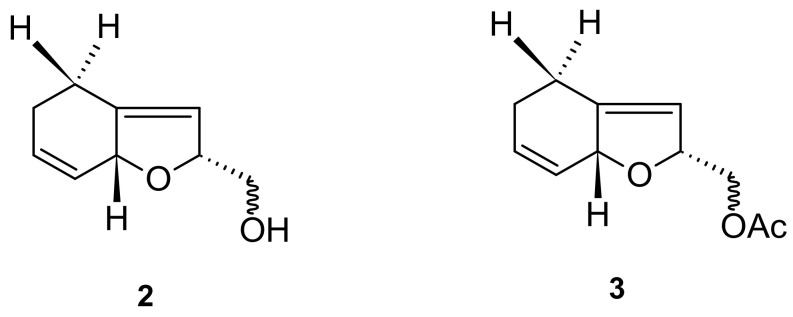
The norisoprenoids (**2** and **3**) from *I. pes-caprae*.

Two norisoprenoids—actinidols 1a (**2**) and 1b (**3**)—were isolated from *I. pes-caprae* oil, as shown in Figure 3 [31].

### 5.3. Phenols

**Figure 4:**
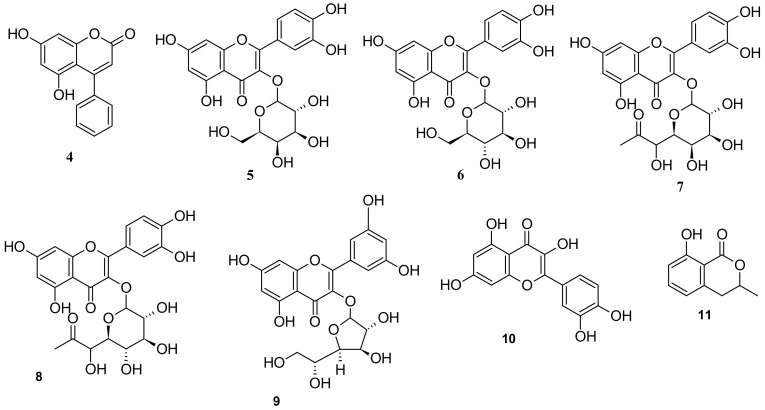
The flavonoids (**4**–**10**) and coumarin (**11**) from *I. pes-caprae*.

Twenty-three phenols (**4**–**26**) have been isolated from *I. pes-caprae*, mostly composed of flavonoids and coumarins. These include seven flavonoids, one coumarin, and other phenolic compounds. The flavonoids and flavonoid glycosides are 5,7-dihydroxy-4-phenyl-2*H*-chromen-2-one (**4**), quercetin 3-*O*-galactoside (**5**), quercetin 3-*O*-glucoside (isoquercetin; **6**), quercetin 3-*O*-acetylgalactoside (**7**), quercetin 3-*O*-acetylglucoside (**8**), quercetin 3-*O*-*β*-d-glucofuranoside (**9**), and quercetin (**10**) [24,32,33]. A coumarin compound, (-) mellein (**11**) has also been isolated from *I. pes-caprae* [34] (Figure 4).

**Figure 5:**
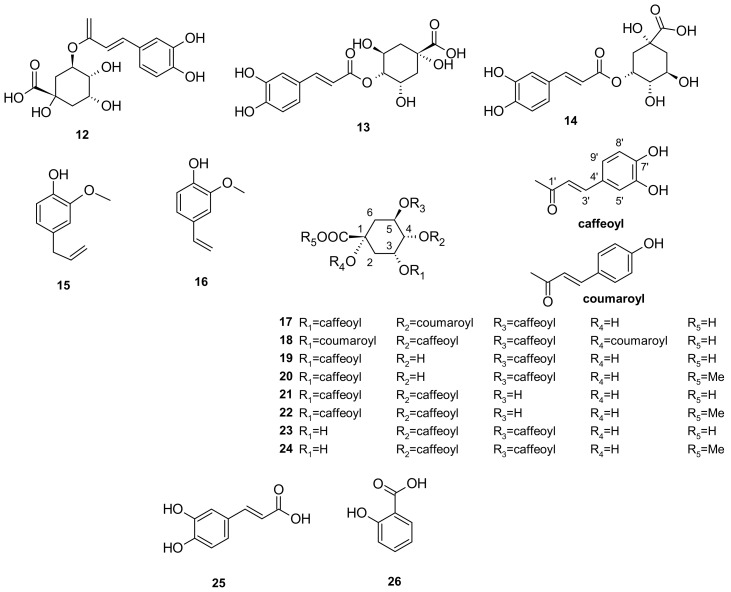
Other phenols (**12**–**26**) from *I. pes-caprae*.

The other phenols identified from *I. pes-caprae* include 3-*O*-caffeoylquinic acid (**12**), 4-*O*-caffeoylquinic acid (**13**), 5-*O*-caffeoylquinic acid (chlorogenic acid; **14**), eugenol (**15**), 4-vinylguaiacol (**16**), the quinic acid esters – 3,5-di-*O*-caffeoyl-4-*O*-coumaroylquinic acid (**17**), 4,5-di-*O*-caffeoyl-1,3-di-*O*-coumaroylquinic acid (**18**), 3,5-di-*O*-caffeoylquinic acid (isochlorogenic acid A; **19**), 3,5-di-*O*-caffeoylquinic acid methyl ester (**20**), 3,4-di-*O*-caffeoylquinic acid (isochlorogenic acid B; **21**), 3,4-di-*O*-caffeoylquinic acid methyl ester (**22**), 4,5-di-*O*-caffeoylquinic acid (isochlorogenic acid C; **23**), and 4,5-di-*O*-caffeoylquinic acid methyl ester (**24**), caffeic acid (**25**), and salicylic acid (**26**) [24,33,34,35]. The chemical structures are listed in Figure 5.

### 5.4. Terpenoids

**Figure 6:**
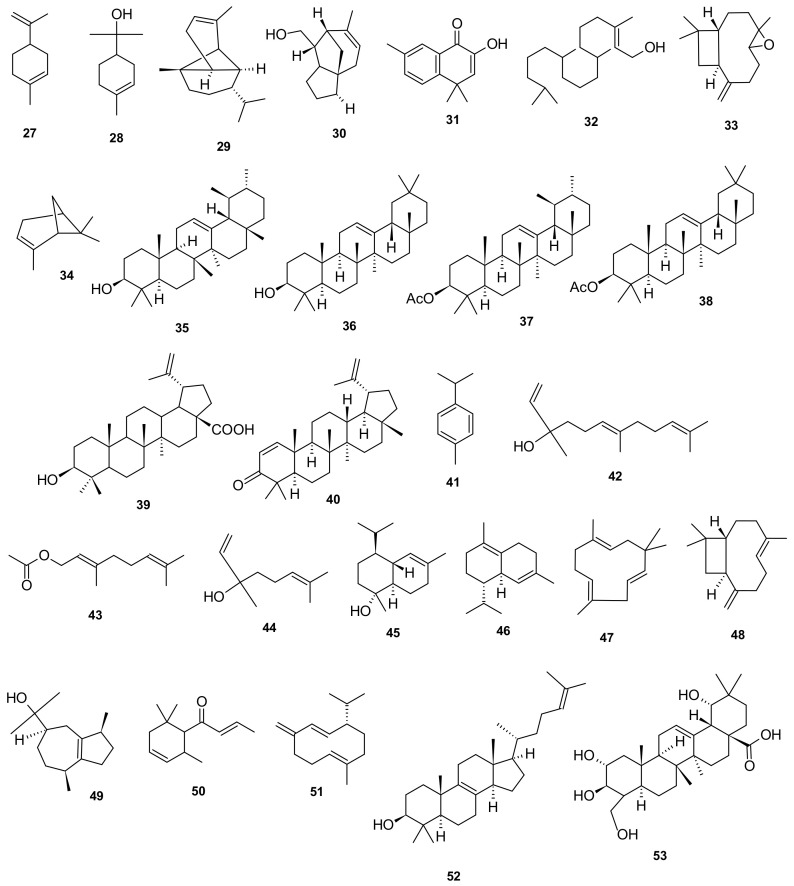
The terpenoids (**27**–**53**) from *I. pes-caprae*.

Twenty-seven terpenoidal compounds (**27**–**53**) have been reported in *I. pes-caprae*: limonene (**27**), *α*-terpineol (**28**), *α*-copaene (**29**), 8-cedren-13-ol (**30**), 2-hydroxy-4,4,7-trimethyl-1(4*H*)-naphthalenone (**31**), *E*-phytol (**32**), caryophyllene oxide (**33**), *α*-pinene (**34**), *α*-amyrin (**35**), *β*-amyrin (**36**) α-amyrin acetate (**37**), *β*-amyrin acetate (**38**), betulinic acid (**39**), glochidone (**40**), *p*-cymene (**41**), (*E*)-nerolidol (**42**), geranyl acetate (**43**), linalool (**44**), α-cadinol (**45**), *β*-caryophyllene (**46**), *δ*-cadinene (**47**), *α*-humulene (**48**), guaiol (**49**), *β*-damascenone (**50**), germacrene D (**51**), lanosterol (**52**), and sericic acid (**53**) [20,24,34,36] (Figure 6).

### 5.5. Steroids

**Figure 7:**
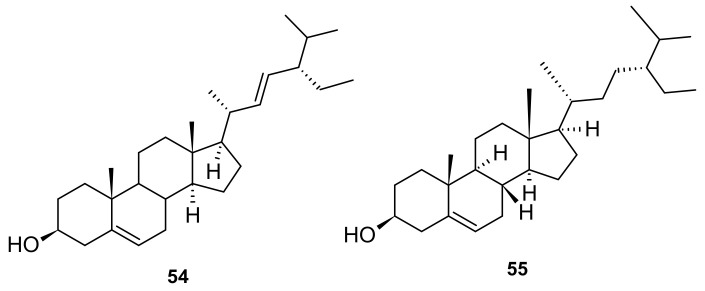
The steroids (**54** and **55**) from *I. pes-caprae*.

Two sterols (**54** and **55**) from *I. pes-caprae* are stigmasterol (**54**) and *β*-sitosterol (**55**) [24]. The chemical structures are shown in Figure 7.

### 5.6. Glycosides

**Figure 8:**
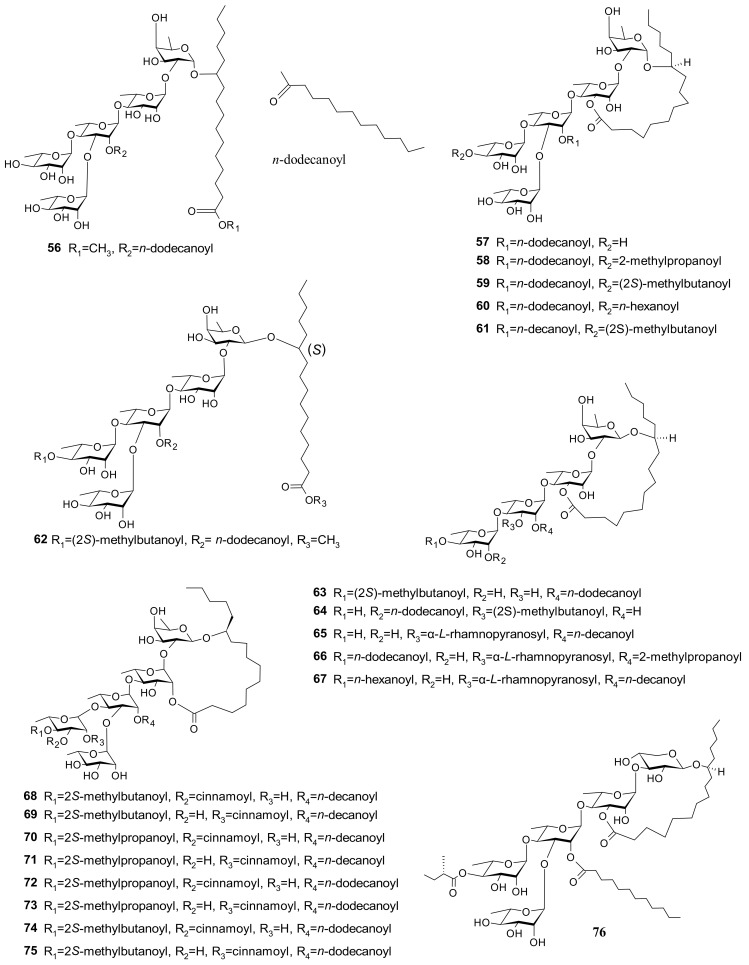

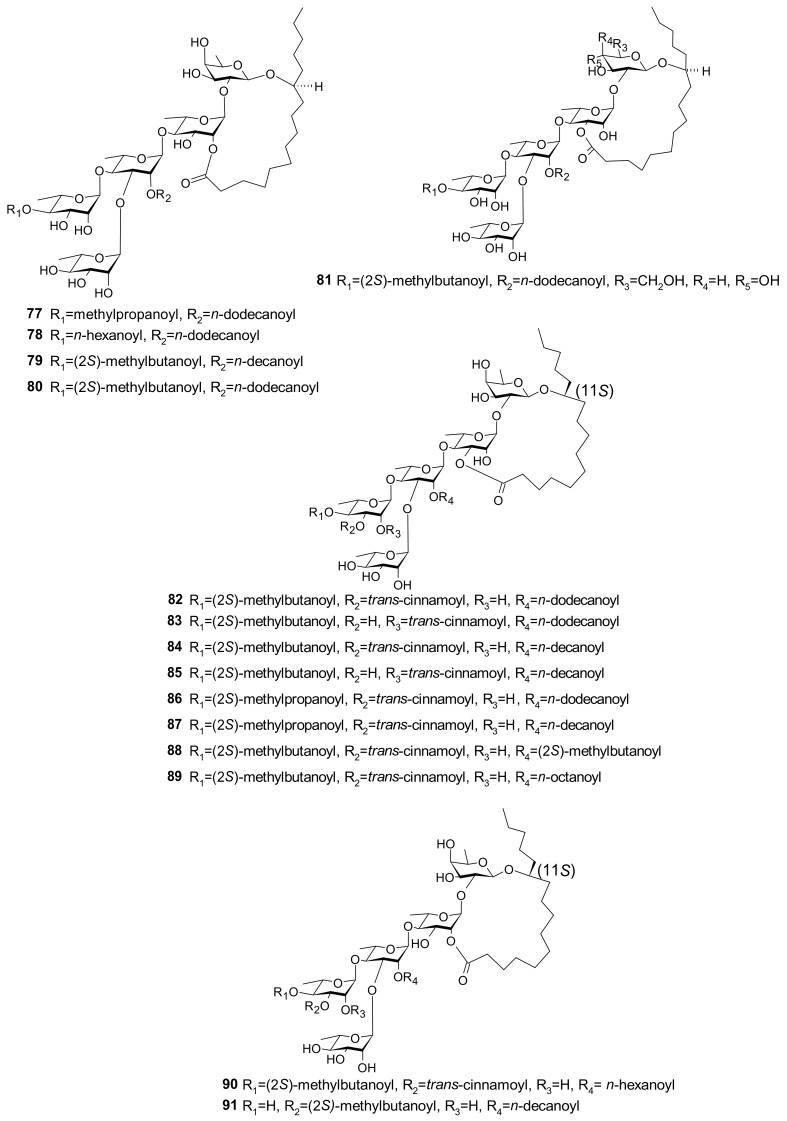
The glycosides (**56**–**91**) from *I. pes-caprae*.

Thirty-six glycosides (**56**–**91**) have been separated and identified from *I. pes-caprae*. These resin glycosides include lipophilic pentasaccharides of jalapinolic acid: pescaproside A (**56**), pescapreins I–IV (**57**–**60**), and stoloniferin III (**61**) [3]; lipophilic oligosaccharides of jalapinolic acid: pescaproside B (**62**), pescapreins V–IX (**63**–**67**) [37], pescapreins X–XVII (**68**–**75**) [38], pescapreins XVIII–XX (**76**–**78**), stoloniferins IX and X (**79** and **80**), and murucoidin VI (**81**) [39]. Pescapreins XXI–XXX (**82**–**91**) have a pentasaccharide core, esterified with different organic acids and lactonized by (11*S*)-hydroxyhexadecanoic acid (jalapinolic acid) to form a macrocyclic lactone [40] (Figure 8).

### 5.7. Other Constituents

**Figure 9:**
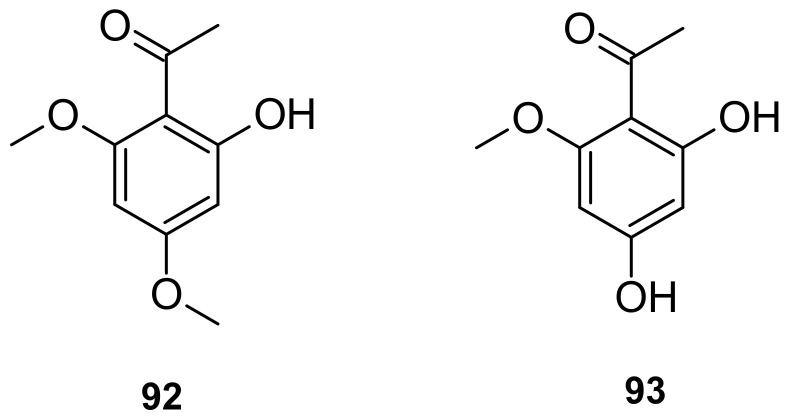
Other compounds (**92** and **93**) from *I. pes-caprae*.

The other compounds isolated from *I. pes-caprae* are xanthoxyline (2′-hydroxy-4′,6′-dimethoxyacetophenone; **92**) and 2,4-dihydroxy-6-methoxyacetophenone (**93**) [24], as listed in Figure 9.

## 6. Bioactivities of *I. pes-caprae*

Several studies have evaluated the extracts, fractions, and isolated compounds from *Ipomoea pes-caprae* for biological activities. Meanwhile, the major activities reported for this plant include antioxidant, anti-inflammatory, antinociceptive, antimicrobial, collagenase inhibitory, antispasmodic, anticancer, antitumor and antiproliferative, and multidrug-resistance efflux inhibitory activities. The secondary metabolites associated with these activities are discussed in the following subsections.

### 6.1. Antioxidant Activity

The presence of excess free radicals in the human body has been implicated in the development of diverse human-related diseases. Secondary metabolites, on the other hand, play important roles by counteracting the effects of free radicals in the body. Such metabolites are often called antioxidants. Many antioxidant substances have been identified from natural sources in past years, including *I. pes-caprae*. Studies on the antioxidant activity of *I. pes-caprae* extracts have shown that it contains phytochemicals with the potential to scavenge free radicals [2]. In a study that evaluated the radical scavenging effects of medicinal halophytes, extract of *Ipomoea pes-caprae* showed strong radical scavenging and reducing power on a 2,2-diphenyl-1-picrylhydrazyl (DPPH) radical with IC_50_ of 32.11 µg/mL, better than the synthetic antioxidants with IC_50_ of 42.15 and 35.24 µg/mL for butylated hydroxyanisole (BHA) and butylated hydroxytoluene (BHT), respectively [41].

Quercetin (**10**), which was previously isolated by Krogh et al. from the methanol extract of *I. pes-caprae* aerial parts, is an effective free-radical flavonoid scavenger [24]. The presence of four hydroxyl (–OH) groups on its benzo-dihydropyran ring helps to eliminate the free radicals produced in the human body and helps the body to maintain a stable state. Low concentrations of **10** inhibited lipid peroxidation by increasing low-density lipoprotein receptor (LDL-R) expression, reducing proprotein convertase subtilisin/kexin type 9 (PCSK9) secretion, and stimulating low-density lipoprotein (LDL) uptake [42]. Majewska et al. observed that **10** has the highest DPPH radical scavenging rate among some tested flavonoids, which included luteolin, rhamnetin, isorhamnetin, and apigenin. Compound **10** had an IC_50_ value of 0.028 µM, stronger than the DPPH radical scavenging activity of standard antioxidants, vitamin C (0.111 µM), and trolox (0.15 µM), used in the experiment [43].

Additionally, the methanol extract of *I. pes-caprae* aerial parts yielded *β*-amyrin (**36**), stigmasterol (**54**), and *β*-sitosterol (**55**) [24]. Compound **36** has also been reportedly isolated from *Symplocos cochinchinensis* leaves where it demonstrated marked antioxidant activity [44]. The IC_50_ value of **36** on superoxide antioxidant activity was found to be 0.190 μM, while BHT and vitamin C had 0.351 and 0.437 μM, respectively [44]. Compound **55**, which was isolated from *Polygonum hydropiper*, also showed strong antioxidant potential by scavenging free radicals of a diverse nature both in vitro and in vivo. The IC_50_ for the DPPH, 2,2′-azino-bis (3-ethylbenzothiazoline6-sulphonic acid) (ABTS), and hydrogen peroxide (H_2_O_2_) radical scavenging property of **55** was calculated as 0.338, 0.289, and 0.675 µM, respectively. Meanwhile, ascorbic acid (positive control) produced IC_50_ values of 0.284, 0.114, and 0.369 µM, respectively [45].

In another study, bioactivity-guided isolation of an acetone extract of the leaves of *I. pes-caprae*, which was collected from the Sarabanga riverbank, Omalur, Salem District, Tamil Nadu, India, afforded a coumarin, identified as 5,7-dihydroxy-4-phenyl-2*H*-chromen-2-one (**4**). Compound **4** expressed significant and dose-dependent activity on DPPH radicals (IC_50_ = 0.032 μM) and hydroxyl radicals (IC_50_ = 0.055 μM) [32]. This is the first report of the antioxidant activities of **4**. However, coumarins have significant therapeutic potential and possess a wide spectrum of biological activities, which is attributable to the free-OH groups at C6, C7, or C8 positions [46].

In a more recent study, quercetin 3-*O*-galactoside (**5**), isoquercetin (**6**), 3-*O*-caffeoylquinic acid (**12**), 4-*O*-caffeoylquinic acid (**13**), chlorogenic acid (**14**), isochlorogenic acid B (**21**), isochlorogenic acid C (**23**), and caffeic acid (**25**) were identified from a methanol fraction of aerial parts of *I. pes-caprae* [33]. Both **12** and **13** showed radical scavenging properties [47]. Previous studies on **5** and **25** also indicated their respective antioxidant properties [48,49]. For instance, compound **6** exhibited high antioxidant activity in a DPPH assay with a half-maximal radical scavenging concentration (RC_50_) of 0.048 µM [50]. Likewise, compounds **14**, **21**, and **23**, which were reportedly isolated from *Bidens pilosa*, showed DPPH scavenging ability with IC_50_ values of 3.29, 3.79, and 10.45 µM using caffeic acid and quercetin as reference compounds [51]. In a different study, compound **25** showed a high DPPH radical scavenging ability with IC_50_ of 0.033 µM, which was greater than the IC_50_ of ascorbic acid (0.245 µM), which served as the positive control [52].

Another antioxidant compound from *I. pes-caprae* is salicylic acid (**26**), an inhibitor of oxidative stress that acts by binding iron (Fe^2+^) that produces OH^−^, peroxy, and alkoxy radicals in the process of lipid peroxidation [53]. Limonene (**27**), *α*-terpineol (**28**), *α*-copaene (**29**), 8-cedren-13-ol (**30**), and *β*-caryophyllene (**46**) are some of the major constituents of the essential oil from the fresh and dried leaves of *I. pes-caprae*, which were collected along Grand Gaube seashore, Mauritius [20]. Compound **27** has been reported in *Citrus medica* for its antioxidant activities [54]. The radical scavenging ability of **46**, which was evaluated by the DPPH and ferric-reducing antioxidant power (FRAP) scavenging test, produced IC_50_ values of 1.25 and 3.23 μM, respectively, compared to ascorbic acid with IC_50_ of 1.5 and 3.8 μM, respectively [55]. Additionally, previous studies have reported the antioxidant potential of **28** and **29** [56,57]. The essential oil of *Peucedanum longifolium* is abundant in **30**, where it exhibited a potent DPPH free-radical scavenging ability and inhibited lipid peroxidation [58]. The list of compounds from *I. pes-caprae* with antioxidant activity is summarized in Table 1.

**Table 1:** Antioxidant activity of *I. pes-caprae* natural products.

Compound	Pharmacological Activities	Reference
**4**	DPPH * scavenging (IC_50_ ** = 0.032 μM) Hydroxyl radical scavenging (IC_50_ = 0.055 μM)	[32]
**6**	DPPH assay (RC_50_ *** = 0.048 µM)	[50]
**10**	DPPH scavenging (IC_50_ = 0.028 µM)	[43]
**14**	DPPH scavenging (IC_50_ = 10.45 µM)	[51]
**21**	DPPH scavenging (IC_50_ = 3.29 µM)	[51]
**23**	DPPH scavenging (IC_50_ = 3.79 µM)	[51]
**25** (Caffeic acid)	DPPH scavenging (IC_50_ = 0.033 µM)	[52]
**36** (*β*-Amyrin)	Superoxide radical scavenging (IC_50_ = 0.190 μM)	[44]
**46** (*β*-Caryophyllene)	DPPH scavenging (IC_50_ = 1.25 μM) FRAP **** scavenging (IC_50_ = 3.23 μM)	[55]
**55** (*β*-Sitosterol)	DPPH scavenging (IC_50_ = 0.338 µM) ABTS ***** scavenging (IC_50_ = 0.289 µM) H_2_O_2_ scavenging (IC_50_ = 0.675 µM)	[45]

* DPPH: 2,2-diphenyl-1-picrylhydrazyl; ** IC_50_: half-maximal inhibitory concentration; *** RC_50_: half-maximal radical scavenging concentration; **** FRAP: ferric-reducing antioxidant power; ***** ABTS: 2,2′-azino-bis (3-ethylbenzothiazoline-6-sulphonic acid).

### 6.2. Anti-Inflammatory Activity

The practice of using plants, their parts, or their extracts for anti-inflammatory purposes has been in existence for a very long time [59]. As previously mentioned, *I. pes-caprae*, for example, is used in Brazil to treat inflammation [4]. The fresh leaves of beach morning glory have been widely used to treat inflammation of toxic effects from jellyfish venoms and dermatitis. During the last decade, in vivo and in vitro studies have led to the discovery of various extracts and compounds with proven anti-inflammatory properties from natural sources [60]. In earlier studies, Pongprayoon et al. reported the inhibitory effect of *Ipomoea pes-caprae* extract (IPA), aspirin, and indomethacin on prostaglandin synthesis [61]. The extract showed a concentration-dependent inhibition of prostaglandin formation in vitro with IC_50_ of 62.3 µg/mL, compared to aspirin and indomethacin with IC_50_ values of 74.8 and 0.30 µg/mL, respectively. The IC_50_ values of the IPA and aspirin are almost equipotent, but lower than indomethacin [34].

Further bioassay-guided separation of IPA yielded (-) mellein (**11**), eugenol (**15**), 4-vinylguaiacol (**16**), and 2-hydroxy-4,4,7-trimethyl-1(4H)-naphthalenone (**31**). Compounds **15** and **16** were the most active with IC_50_ values of 9.2 and 18 µM, respectively, while the IC_50_ values for compounds **11** and **31** were 230 and 340 µM, respectively. The inhibitory effect of compounds **11**, **15**, **16**, and **31** on prostaglandin formation partly explains the observed anti-inflammatory effect of IPA [34]. This represents the only report of the isolation of **31** as a natural product, although previously reported by Davis et al. as a minor product while investigating the biogenesis of tobacco isoprenoids [62]. Generally, phenolic antioxidants act by inhibiting tumor necrosis factor-α (TNF-α)-induced nuclear factor-kB (NF-κB) activation and by blocking cyclooxygenase (COX)-2 expression in lipopolysaccharide (LPS)-stimulated macrophages. Further studies have shown that **15** effectively modulates lung inflammation and remodeling in an in vivo acute lung injury model, by inhibiting TNF-α release and NF-κB activation [63].

A continued effort to isolate secondary metabolites through bioactivity-guided fractionation of the IPA oil also led to the isolation of two diastereomeric compounds, the actinidols la (**2**) and lb (**3**). Although, actinidols have been previously isolated from *Vitis vinifera* and *Acrinidia poly-gama*. Compounds **2**, **3**, **11**, **15**, and **31** and another isolated compound, *E*-phytol (**32**), were assayed for anti-inflammatory activity in an in vivo model of acute inflammation ethyl phenylpropiolate (EPP)-induced edema. These compounds inhibited edema formation in a dose-dependent manner. Compounds **2** and **3** (1.0 mg/ear) produced 35% inhibition, compound **11** (0.6 mg/ear) produced 37% inhibition, compound **15** (1.0 mg/ear) produced 38% inhibition, compound **31** (0.6 mg/ear) produced 30% inhibition, and compound **32** (1.0 mg/ear) produced 47% inhibition, respectively, after 1 h. The controls, oxyphenbutazone (0.6 mg/ear) and papaverine (0.6 mg/ear), produced 41 and 43% inhibition, respectively. The study showed that IPA oil comprises several anti-inflammatory compounds. Compounds **11**, **15**, and **31** might decrease prostaglandin formation, while **32** might reduce vascular leakage by inhibiting the contraction of endothelial cells [36].

Quercetin (**10**), previously isolated by Krogh et al. from the methanol extract of aerial parts of *I. pes-caprae* [24], is a known anti-inflammatory flavonoid. It has shown significant anti-inflammatory activities in human and animal cell types. Experiments conducted by Li et al. on animal models revealed the ability of quercetin to inhibit LPS-induced TNF-α production in macrophages and human lung cancer A549 cell lines [64]. Additionally, compound **10** can inhibit the COX and lipoxygenase (LOX) enzymes that produced inflammation [42]. The evaluation of the inhibitory effect of **10**, engeletin, and astilbin, isolated from *Smilax corbularia* on LPS-induced nitric oxide (NO) production, TNF-α, and prostaglandin E2 (PGE2) release in RAW 264.7 cells revealed that **10** showed the best inhibitory effect on TNF-α and NO production with IC_50_ values of 4.14 and 37.1 µM, respectively. Interestingly, compound **10** showed higher IC_50_ values for the inhibition of NO production and TNF-α release than indomethacin, which was used as the positive control, except for PGE2 release. The inhibitory effects of indomethacin on NO, TNF-α, and PGE2 release were 56.8, 143.7, and 2.8 µM, respectively [65].

Both caryophyllene oxide (**33**; sesquiterpenoid oxide) and α-pinene (**34**; monoterpene)—major components of the essential oil of fresh and dried leaves of *I. pes-caprae*—have been reported for their anti-inflammatory activities in other studies. Compound **33**, previously isolated from *Annona squamosa* bark extract, exhibited significant anti-inflammatory activities [66]. From the acetic acid-induced writhing test in mice, **33** inhibited the writhing response by 75.19% at 25 mg/kg body weight, almost to the same degree as aspirin (74.41%) at 100 mg/kg body weight [66]. Other studies have also confirmed the anti-inflammatory potential of **34** [67].

α-Amyrin (**35**) and β-amyrin (**36**), along with their acetates, α-amyrin acetate (**37**) and β-amyrin acetate (**38**), have shown anti-inflammatory activities in previous studies [68,69]. In the study of Gupta et al., the effects of **37** and **38** were studied on granulation tissue formation by the cotton-pellet implantation method in albino rats. The results showed that 4 mg/100 g i.p. of **37** and **38** resulted in 19.1 and 43.6% anti-inflammation, respectively, while 1 mg/100 g i.p. hydrocortisone (standard drug) produced 31.3% anti-inflammation [68]. Betulinic acid (**39**), stigmasterol (**54**), and β-sitosterol (**55**) have all been reported in the literature for their anti-inflammatory properties [24,69]. The anti-inflammatory properties of these metabolites are summarized in Table 2.

**Table 2:** Anti-inflammatory activity of *I. pes-caprae* natural products.

Compound	Pharmacological Activities	Reference
**2** (Actinidol la) **3** (Actinidol lb)	1.0 mg/ear produced 35% inhibition of oedema formation	[31]
**10**	Inhibitory effect on NO * production IC_50_ ** = 37.1 µM Inhibitory effect on TNF-α *** production with IC_50_ = 4.14 µM	[65]
**11** [(-) Mellein]	Inhibition of prostaglandin synthesis IC_50_ = 340 µM 1.0 mg/ear produced 37% inhibition of oedema formation	[31,34]
**15** (Eugenol)	1.0 mg/ear produced 38% inhibition of oedema formation. Inhibition of prostaglandin synthesis IC_50_ = 9.2 µM	[31,34]
**16**	Inhibition of prostaglandin synthesis IC_50_ = 18 µM	[34]
**31**	0.6 mg/ear produced 30% inhibition of oedema formation. Inhibition of prostaglandin synthesis IC_50_ = 230 µM	[31,34]
**32** (*E*-phytol)	1.0 mg/ear produced 47% inhibition of oedema formation	[31]
**33** (Caryophyllene oxide)	Inhibited writhing response by 75.19% at 25 mg/kg body weight	[66]
**37** (*α*-Amyrin acetate)	4 mg/100g i.p. **** produced 19.1% inhibition	[68]
**38** (*β* -Amyrin acetate)	4 mg/100g i.p. produced 43.6% inhibition	[68]

* NO: nitric oxide; ** IC_50_: half-maximal inhibitory concentration; *** TNF-α: tumor necrosis factor-α; **** i.p.: intraperitoneal.

### 6.3. Antinociceptive Activity

Another traditional importance of beach morning glory is for treating dolorous processes. Pain is an unpleasant sensation associated with tissue damage. Generally, drugs used to treat pain are in high demand worldwide, either as opiates or non-steroidal anti-inflammatory drugs (NSAIDs). Nowadays, much attention has been paid to screening analgesics from natural origins due to their lower side effects compared to opiates and NSAIDs [70]. Among such studies, Maria De Souza et al. examined the antinociceptive actions of a methanol extract and fractions from *I. pes-caprae* aerial parts [71]. The methanol extract produced an half-maximal infective dose (ID_50_) of 33.8 mg/kg i.p. (writhing test) and also inhibited both the first and second phases of pain (neurogenic and inflammatory) in the formalin test, with ID_50_ values of 37.7 and 12.5 mg/kg i.p., respectively [71].

Krogh et al. described the isolation of antinociceptive-active compounds from the methanol extract of *I. pes-caprae* [24]. Among the identified metabolites, isoquercetin (**6**), *α*- and *β*-amyrin acetate (**37** and **38**), betulinic acid (**39**), and glochidone (**40**) produced significant antinociceptive properties of 34.5, 54.4, 88.1, and 75.5% inhibition, respectively, against aspirin with 35.0% inhibition at 10 mg/kg i.p. in an acetic acid-induced abdominal constriction test. These findings supported the widespread use of *I. pes-caprae* to treat dolorous conditions [24]. Both **37** and **38** are derivatives of *α*-amyrin (**35**) and *β*-amyrin (**36**), respectively. Compounds **35** and **36** have been previously isolated from the resin of *Protium kleinii* where they showed significant antinociceptive activity against acetic acid-induced visceral pain in mice. The study suggested that the mechanism of action involves the inhibition of protein kinase A- and C-sensitive pathways [72]. In addition to the antinociceptive activity of **39** above, studies on **39** using the abdominal contortions model induced by acetic acid also proved its significant antinociceptive activity[73].

*α*-Terpineol (**28**), *p*-cymene (**41**), and (*E*)-nerolidol (**42**) are some of the major essential oils identified from the fresh and dried *I. pes-caprae* leaves in Mauritius [20]. These oils have shown considerable antinociceptive actions in the literature. For example, the evaluation of the antinociceptive actions of three monoterpenes by acetic acid-induced writhing and formalin-induced nociceptive tests in mice showed that **41** has the best in vivo and in vitro antinociceptive effect compared to (+) camphene and geranyl acetate in experimental models, reducing nociception in both phases of the formalin test [74]. The antinociceptive activities of the monoterpene **28** and the sesquiterpene alcohol **42** have been reported in other works [19,57]. Sericic acid (**53**), which was previously isolated from *Vochysia divergens* Pohl., also possesses antinociceptive properties [24]. The antinociceptive compounds identified in beach morning glory are listed in Table 3.

**Table 3:** Antinociceptive activity of *I. pes-caprae* natural products.

Compound	Pharmacological Activities	Reference
**6**	10 mg/kg i.p. * inhibited constriction by 34.5%	[24]
**37** (α-Amyrin acetate) **38** (β-Amyrin acetate)	10 mg/kg i.p. inhibited constriction by 54.4%	
**39** (Betulinic acid)	10 mg/kg i.p. inhibited constriction by 88.1%	
**40** (Glochidone)	10 mg/kg i.p. inhibited constriction by 75.5%	

* i.p: intraperitoneal.

### 6.4. Antimicrobial Activity

Several studies have been conducted on *I. pes-caprae*, confirming its use in folklore medicine to treat diseases caused by microorganisms. For instance, the evaluation of the antibacterial activity of different solvent extracts of *I. pes-caprae* whole plant against some Gram-positive and Gram-negative bacteria revealed interesting activities in the ethyl acetate and the acetone extract only with minimum inhibitory concentrations (MIC) of 12.5 and 25 mg/mL, respectively, against *Staphylococcus* [75].

As previously mentioned, (-) mellein (**11**) was isolated from IPA oil, which was obtained from the steam distillation and extraction of dried *I. pes-caprae* leaves with petroleum ether [31]. Although this is not the first report of isolating this metabolite from natural sources, as it had been previously isolated from *Aspergillus melleus* and other microbial sources, it possesses antibacterial activity [34]. The diterpene, *E*-phytol (**32**), obtained from the bioassay-guided fractionation of IPA by Pongprayoon et al. was evaluated by the (3-[4,5-dimethylthiazol-2-yl]-2,5 diphenyl tetrazolium bromide) (MTT) assay against two bacterial and fungal strains—*Escherichia coli*, *Candida albicans*, *Aspergillus niger*, and *Staphylococcus aureus*. The half-maximal minimum inhibitory concentrations (MIC_50_) of **32** against *E. coli*, *C. albicans*, and *A. niger* was 0.219 µM, and > 3.37 µM against *S. aureus* in a dose- and time-dependent manner [76].

Alagesan et al. isolated 5,7-dihydroxy-4-phenyl-2*H*-chromen-2-one (**4**) from the acetone extract of *I. pes-caprae* [32]. Compound **4** displayed maximum growth inhibition against *E. coli* and *Shigella flexneri*. This is the first report of the antimicrobial activity of this coumarin compound. Based on the previous studies, the carbon-7 (C7) free –OH group of coumarins is essential for inhibiting the growth of Gram-positive bacteria. This explains the observed activity of **4** since it contains free –OH groups at the carbon-5 (C5) and C7 positions [32]. Quercetin (**10**) is a broad-spectrum antibacterial and has significant fungal inhibitory activity. The antimicrobial activity of quercetin is achieved through diverse mechanisms of action, which include the prevention of bacterial adhesion, the inhibition of quorum-sensing pathways, the destruction or change of the plasma membrane, the inhibition of efflux pumps, affecting protein synthesis and expression, and the blockage of nucleic acid synthesis [42].

*β*-Sitosterol (**55**), which was identified from the methanol extract of *I. pes-caprae* aerial parts, is known for its antimicrobial properties. Another study that evaluated the effect of **55** on *Escherichia coli, Pseudomonas aeruginosa, Staphylococcus aureus*, and *Klebsiella pneumoniae* found that **55** (0.048 μM) produced an antimicrobial activity almost equivalent to gentamicin (0.042 μM) [77].

Among the compounds identified by Gonçalves et al. from the methanolic fraction of a hydroalcoholic extract of *I. pes-caprae* aerial parts [33], chlorogenic acid (**14**) and caffeic acid (**25**) have been identified as antimicrobial agents [78,79]. Toyama et al. isolated **14** from *Baccharis oxyodonta*, which induced the destruction of many bacteria cells [80]. Zhang et al. stated that **14** acts by binding and disrupting the outer membrane, exhausting the intracellular potential, and therefore releasing the cytoplasmic macromolecules, which in turn leads to cell death [81]. In a different study, **14** effectively inhibited both Gram-positive and Gram-negative bacteria with MIC values of 0.226, 0.057, and 0.113 μM against *E. coli*, *Shigella dysenteriae*, and *Salmonella typhimurium*, respectively, while the MIC values were 0.057, 0.113, and 0.113 μM for *Streptococcus pneumoniae*, *Staphylococcus aureus*, and *Bacillus subtilis*, respectively [78]. On the other hand, compound **25**, which is widely distributed in plant tissues, exhibits in vitro antibacterial activity [48]. In a study that evaluated the activity of **25** towards the staphylococcal strains using the standard microdilution liquid method, *S. aureus* ATCC 25293 was one of the most susceptible strains to **25** with a MIC value of 1.421 μM [79].

Marie et al. identified limonene (**27**), *α*-pinene (**34**), (*E*)-nerolidol (**42**), linalool (**44**), *α*-cadinol (**45**), *β*-caryophyllene (**46**), *δ*-cadinene (**47**), and guaiol (**49**) as components of the essential oil of the leaves of *I. pes-caprae* [20]. These essential oils have been extensively studied and shown to possess important antimicrobial activities. Compounds **27** and **44** show antibacterial, antiviral, and antifungal properties [54]. Compound **45** is an antifungal sesquiterpene [82]. Compound **46** has been reported to be a responsible component for the antimicrobial activity of *Aquilaria crassnia*. It was demonstrated that **46** significantly inhibited the growth of *S. aureus*, although ineffective against *K. pneumoniae*. The MIC values of **46** and kanamycin (standard drug) against *S. aureus* are 3 and 8 µM, respectively [55]. Compound **49** is sesquiterpene alcohol found in many medicinal plants with proven antibacterial activity [83]. The antimicrobial properties of **34** and **42** have also previously been reported [19,67].

Sericic acid (**53**), another known metabolite from *I. pes-caprae*, demonstrated strong antifungal activity from studies conducted on *Terminalia sericea* and *Vochysia divergens* [24,84]. Against *T. sericea* (MIC of 0.135 µM), compound **53** had better activity than the standard drug clotrimazole (MIC of 0.261 µM) [84]. This finding correlates with Krogh et al. on the antifungal activity of **53** isolated from *I. pes-caprae* [24]. This may be due to the presence of conjugated carbons, some phenolic, hydroxyl, and carboxyl groups, and the number of acceptor atoms of hydrogen bonds in the compound, which are important structural descriptors for the antimicrobial activity of terpenes [85]. Xanthoxyline (**92**) is the main antifungal constituent isolated from *Sebastiania schottiana* [86]. It is also active against *Cryptococcus neoformans and Aspergillus fumigatus* with MICs of 0.255 and 0.382 µM, respectively [86]. The antimicrobial compounds from *I. pes-caprae* are summarized in Table 4.

**Table 4:** Antimicrobial activity of *I. pes-caprae* natural products.

Compound	Pharmacological Activities	Reference
**14** (Chlorogenic acid)	MIC * value of 0.057 µM against *Shigella dysenteriae* and MIC value of 0.113 µM against *Staphylococcus aureus* (Antibacterial)	[78]
**25** (Caffeic acid)	MIC value of 1.421 μM against *S. aureus* ATCC 25293 (antibacterial)	[79]
**32** (*E*-Phytol)	MIC_50_ ** value of 0.219 µM against *Escherichia coli* and MIC value of 3.37 µM against *S. aureus* (antibacterial) MIC_50_ value of 0.219 µM against *Candida albicans* and *Aspergillus niger* (antifungal)	[76]
**46** (*β*-Caryophyllene)	MIC value of 3 μM against *S. Aureus* (antibacterial)	[55]
**53** (Sericic acid)	MIC value of 0.135 µM against *C. albicans* and *Cryptococcus neoformans* (antifungal)	[84]
**55** (*β*-Sitosterol)	0.048 μM produced inhibition zones of 14 mm (*E. coli*), 13 mm (*S. aureus*), 11 mm (*Pseudomonas aeruginosa*), and 10 mm (*Klebsiella pneumoniae*) (antibacterial)	[77]
**92** (Xanthoxyline)	MIC value of 0.255 µM against *C. neoformans* and MIC value of 0.382 µM against *Aspergillus fumigatus* (antifungal)	[86]

* MIC: minimum inhibitory concentration; ** MIC_50_: half-maximal minimum inhibitory concentration.

### 6.5. Collagenase Inhibitory Activity

The depletion of collagen by collagenase enzymes is involved in various human pathologies, such as arthritis, cancer, cardiovascular diseases, and neurodegenerative diseases. This makes collagenase an important target for the pharmaceutical and cosmetic industry [87]. In a study conducted by Teramachi et al. to evaluate the collagenase inhibitory activity of the fractions from the methanolic extract of *I. pes-caprae* leaves from Khon Kaen, Thailand, the ethyl acetate-soluble fraction was the most active compared to others, which included *n*-hexane, *n*-butanol, and water-soluble fractions[35]. From the ethyl acetate fraction, two new quinic acid esters, 3,5-di-*O*-caffeoyl-4-*O*-coumaroylquinic acid (**17**) and 4,5-di-*O*-caffeoyl-1,3-di-*O*-coumaroylquinic acid (**18**), and six known quinic acid esters (caffetannins, **19**–**24**) were isolated. The methyl esters (**20**, **22**, and **24**) were formed during extraction with methanol. Compounds **19**, **21**, and **23** have the trivial names of isochlorogenic acid A, B, and C, respectively. The collagenase inhibition activity for compounds **17**–**24** yielded IC_50_ values of 19.1, 14.2, 23.6, 5.8, 31.7, 16.2, 37.2, and 26.6 µM, respectively (Table 5). Phosphramidon with IC_50_ of 0.6 µM was used as the positive control. The methyl ester **20** produced the best activity. In addition, a cytotoxicity test conducted for compounds **17**, **18**, **23**, and **24** against Jurkat human T-cell leukemia cells showed that the compounds are almost non-cytotoxic (IC_50_ value > 35 µM/mL). In the same study, caffeic acid (**25**) showed weak collagenase inhibition (IC_50_ = 82.7 µM), whereas quinic acid was not active (IC_50_ > 500 µM) [35].

**Table 5:** Collagenase inhibitory activity of *I. pes-caprae* natural products.

Compound	Collagenase Inhibitory Activity IC_50_ * Value (µM)	Reference
**17**	19.1	[35]
**18**	14.2	
**19** (Isochlorogenic acid A)	23.6	
**20**	5.8	
**21** (Isochlorogenic acid B)	31.7	
**22**	16.2	
**23** (Isochlorogenic acid C)	37.2	
**24**	26.6	
**25** (Caffeic acid)	82.7	

* IC_50_: half-maximal inhibitory concentration.

### 6.6. Antispasmodic Activity

Antispasmodics are widely used to relieve breathing problems, muscle spasms, gastrointestinal cramps, and movement disorders associated with impaired contraction and smooth-muscle relaxation. The antispasmodic effect of several medicinal plants has been reported, including *I. pes-caprae*, due to tolerance and potential adverse effects associated with present drugs [88]. The evaluation of the antispasmodic properties of a lipophilic IPA obtained from *I. pes-caprae* collected along Bangsaen seashores, Thailand, showed a 23% inhibition at 31 µg/mL, which indicated the antispasmodic potential of the plant. [36]. As a continuation, both *E*-phytol (**32**) and *β*-damascenone (**50**) were obtained from the bioassay-guided fractionation of IPA by Pongprayoon et al. and were identified as possessing antispasmodic activity [36]. The result obtained showed that 0.105 µM of **32**, 0.163 µM of **50**, and 0.091 µM papaverine produced 41, 45, and 39% inhibitions, respectively, on submaximal contractions of guinea-pig ileal smooth muscle induced by histamine. The antispasmodic potencies of **32** and **50** were similar to papaverine—a spasmolytic agent. The interference with the contraction of the endothelial cell plays a role in IPA’s observed anti-inflammatory activity [2]. Furthermore, compound **32** decreased vascular leakage by inhibiting the contraction of endothelial cells. Severe vascular contraction has been implicated in dermatitis caused by a jellyfish sting, leading to local vascular insufficiency and gangrene. As such, compounds with direct vasodilating activity, such as papaverine, have been recommended to treat toxin-induced dermatitis. The use of IPA is effective to treat toxin-induced dermatitis and has a non-specific antispasmodic action. Therefore, compounds **32** and **50** may be responsible for IPA’s effectiveness toward jellyfish poison by inhibiting vascular smooth-muscle-cell contractions [36].

Isochlorogenic acid A (**19**), isolated from the ethyl acetate soluble fraction of a methanol extract of *I. pes-caprae* leaves also presents significant antispasmodic activities [89]. Xanthoxyline (**92**) is the main antispasmodic component isolated from *Sebastiania schottiana* Muehl. Arg. dried stem and leaves. From the study, the in vitro IC_50_ of xanthoxyline against acetylcholine-induced contraction in guinea-pig ileum was 47 µM [90] (for details, see Table 6).

**Table 6:** Antispasmodic activity of *I. pes-caprae* natural products.

Compound Name	Pharmacological Activities	Reference
**32** (*E*-Phytol)	0.105 µM produced 41% inhibition on submaximal contractions of guinea-pig ileal smooth muscle	[36]
**50** (*β*-Damascenone)	0.163 µM produced 45% inhibitions respectively of submaximal contractions of guinea-pig ileal smooth muscle	
**92** (Xanthoxyline)	Inhibition of acetylcholine-induced contraction in guinea-pig ileum (IC_50_ * = 47 µM)	[90]

* IC_50_: half-maximal inhibitory concentration.

### 6.7. Anticancer, Antitumor, and Antiproliferative Activities

The increase in the incidence of various types of cancer creates a need for new anticancer drugs. As a result, numerous anticancer compounds from plant sources have been/are being examined on various cancer cells and experimental animals, leading to a dynamic increase in newly discovered natural compounds with potent anticancer activities [91]. For instance, a comprehensive study revealed that *I. pes-caprae* methanol extract exhibited a better antitumor effect against melanoma than aqueous and petroleum ether extracts and swaras (fresh juice extracts), but lower than dacarbazine. The tumor volume inhibition of the extract on melanoma cells was significant (*p* < 0.01) and concentration dependent [28].

Six lipophilic glycosides have been obtained from the hexane-soluble extract of *I. pes-caprae* herbal drugs (aerial parts), which was purchased at a local healthfood store in Mexico City, through preparative-scale high-performance liquid chromatography (HPLC) separation. These glycosides are pescaproside A (**56**) and pescapreins I–IV (**57**–**60**), as well as stoloniferin III (**61**). The cytotoxicity of compounds **56**–**61** was tested against four human cancer cell lines—nasopharyngeal, squamous cell cervical, ovarian, and colon carcinomas—and a weak cytotoxicity with median effective dose (ED_50_) range of 5–20 μg/mL was observed [3].

The antiproliferative activity of 5,7-dihydroxy-4-phenyl-2*H*-chromen-2-one (**4**) on human colon cancer cells HCT-116 was examined by MTT assay. In the study, 24 h of treatment of the HCT-116 cell with compound **4** decreased cell viability by about 30% (IC_50_ = 0.055 μM). Molecular docking studies revealed that **4** is a good angiopoietin-2 inhibitor, thus confirming the significant antiproliferative activity of this secondary metabolite [32]. Quercetin (**10**) is another compound from *I. pes-caprae* whose antitumor potential has been proven in vivo and in vitro through various mechanisms from different studies. For instance, an evaluation of the effects of different concentrations of **10** on human leukemia cell lines HL-60 revealed that this compound inhibited HL-60 cell growth in a concentration-dependent manner, with an IC_50_ value of about 7.7 μM after 96 h of treatment [92]. In another study, Chou et al. stated that **10** affected the regulation of tumor protein p53-related pathways in the tumor cell cycle by inducing endoplasmic reticulum (ER) stress, promoting p53 release, and inhibiting cyclin A, cyclin B, and cyclin dependent kinase-2 (CDK2) activities, thus causing the stagnation of MCF-7 breast cancer cells in the S phase [93].

Stigmasterol (**54**) is another anticancer and antiproliferative compound identified from the methanol extract of the fresh aerial part of *I. pes-caprae* [24]. Compound **54** inhibited the proliferation and colony formation potential of gastric cancer cells (IC_50_ = 15 μM) by cell counting kit-8 (CCK-8) assay with no effect observed in untreated cells. Furthermore, 30 μM of **54** increased the percentage of apoptotic cells in gastric cancer SNU-1 cells from 1.75 to 43.66%. The apoptotic effects of **54** were also found to be concentration dependent. These results indicate the potential of **54** as an antiproliferative and anticancer metabolite [94]. Both *α*-amyrin (**35**) and *β*-sitosterol (**55**) have in vitro cytotoxic activities. In a study, the pentacyclic triterpene **35** was tested for its antiproliferative activity against cancer cell lines. The result showed an IC_50_ value of 0.022 and 0.052 μM against A549 and human ovarian cancer cell lines A2780, in contrast to 0.00036 and 0.00058 μM for etoposide, respectively [95]. On the other hand, compound **55** remarkably inhibited the proliferation of human liver cancer cell lines HepG2 and human liver carcinoma cell lines Huh7 cells in a dose-dependent manner with IC_50_ values of 0.017 and 0.021 µM, respectively, indicating that 55 exhibited cytotoxic effects through the induction of apoptosis and activation of caspase-3 and -9 in these cells. Camptothecin (positive control) produced similar results with IC_50_ values of 0.011 and 0.012 µM for HepG2 and Huh7, respectively [96].

The essential oils limonene (**27**), *α*-terpineol (**28**), *α*-pinene (**34**), (*E*)-nerolidol (**42**), and linalool (**44**), identified from *I. pes-caprae* leaves, have anticancer and antitumor properties [57,97,98]. In a study that evaluated the ability of certain essential oils, including **27**, **28**, **34**, and **44**, to inhibit the proliferation of A549 cells in an MTT assay, using untreated cells as control. The IC_50_ values that produced observed inhibitory effects among these four essential oils are—**44** (0.919 μM), **28** (0.333 μM), **27** (0.162 μM), and **34** (0.162 µM). Their inhibitory activities were concentration dependent [98]. *β*-Caryophyllene (**46**) is the active anticancer compound of *Aquilaria crassnia* [55]. Compound **46** showed selective anti-proliferative action against human colorectal carcinoma cell lines HCT116 (IC_50_ = 19 µM) and human pancreatic cancer cell lines PANC-1 (IC_50_ = 27 µM) cells, with little toxicity against normal cells. The positive controls used were 5-fluorouracil (HCT116, IC_50_ = 12.7 µM) and betulinic acid (PANC-1, IC_50_ = 19.4 µM) [55]. Furthermore, betulinic acid (**39**) has a marked antitumor therapeutic effect on melanoma cells and several solid tumor types, including glioblastoma, lung carcinoma, breast carcinoma, colorectal carcinoma, and prostate carcinoma [99]. The investigation of the effect of 2,4-dihydroxy-6-methoxyacetophenone (**93**), which was also isolated from *Euphorbia tibetica* on human lung cancer cell A549, indicated that 0.275 µM inhibited the growth of the cancer cell by 45.20% [100]. The metabolites identified for anticancer, antitumor, and antiproliferative activities from *I. pes-caprae* are summarized in Table 7.

**Table 7:** Anticancer, antitumor, and antiproliferative activities of *I. pes-caprae* natural products.

Compound	Pharmacological Activities	Reference
**4**	Decreased HCT116 * cell viability up to 30% after 24 h of treatment (IC_50_ ** = 0.055 μM)	[32]
**10**	Inhibition of the growth of HL-60 *** cells (IC_50_ = 7.7 μM)	[92]
**27** (Limonene)	Inhibition of the proliferation of A549 **** cells (IC_50_ = 0.162 μM)	[98]
**28** (*α*-Terpineol)	Inhibition of the proliferation of A549 cells (IC_50_ = 0.333 μM)	[98]
**34** (*α*-Pinene)	Inhibition of the proliferation of A549 cells (IC_50_ = 0.162 µM)	[98]
**35** (*α*-Amyrin)	Inhibition of the proliferation of A549 (IC_50_ = 0.022) and A2780 ***** cell lines (IC_50_ = 0.052 μM)	[95]
**44** (Linalool)	Inhibition of the proliferation of A549 cells (IC_50_ = 0.919 μM)	[98]
**46** (*β*-Caryophyllene)	Selective anti-proliferative effect against HCT116 (IC_50_ = 19 µM) and PANC-1 ****** (IC_50_ = 27 µM)	[55]
**54** (Stigmasterol)	Inhibition of proliferation and colony formation of gastric cancer SNU-1 cells ******* (IC_50_ = 15 µM) 30 μM increased the percentage of apoptotic cells in gastric cancer SNU-1 cells from 1.75 to 43.66%	[94]
**55** (*β*-Sitosterol)	Inhibition of the proliferation of HepG2 ******** (IC_50_ = 0.017 μM) and Huh7 ********* cells (IC_50_ = 0.021μM)	[96]
**56** (Pescaproside A) **57** (Pescaprein I) **58** (Pescaprein Ⅱ) **59** (Pescaprein III) **60** (Pescaprein IV) **61** (Stoloniferin III)	Weak cytotoxicity against nasopharyngeal, colon, squamous cell cervical, and ovarian carcinomas (ED_50_ ********** = 5–20 µg/mL)	[3]
**93**	45.2% inhibition of the growth of human lung cancer cell A549 at 0.275 µM	[100]

* HCT116: human colorectal carcinoma cell lines; ** IC_50_: half-maximal inhibitory concentration; *** HL-60: human leukemia cell lines; **** A549: human lung cancer cell lines; ***** A2780: human ovarian carcinoma cell lines; ****** PANC-1: human pancreatic cancer cell lines; ******* SNU-1: human stomach cancer cell lines; ******** HepG2: human liver cancer cell lines; ********* Huh7: human liver carcinoma cell lines; ********** ED_50_: median effective dose.

### 6.8. Multidrug-Resistance Efflux-Inhibiting Activity

Resin glycosides show the ideal structural features associated with multidrug-resistant efflux-pump substrates [101]. Pescapreins II and III (**58** and **59**), stoloniferin III (**61**), pescapreins XVIII–XX (**76**–**78**), stoloniferins IX and X (**79** and **80**), and murucoidin VI (**81**), which were isolated from the chloroform extract of *I. pes-caprae* whole plant have been tested in vitro for their antibacterial and resistance-modulating activity against *S. aureus* strains possessing multidrug-resistance (MDR) efflux mechanisms [39]. These resin glycosides potentiated norfloxacin effect against the NorA over-expressing *S. aureus* strain, SA-1199B, by 4-fold (from 32 μg/mL to 8 μg/mL) at a concentration of 25 μg/mL. Meanwhile, reserpine (positive control) increased norfloxacin action against SA-1199B by 4-fold (from 32 μg/mL to 8 μg/mL) at 20 μg/mL [39]. No report of the multidrug-resistance efflux inhibition was found for pescaproside B (**62**) and pescapreins V–IX (**63**–**67**), which were identified from hexane extract of *I. pes-caprae* whole plant [37] and pescapreins X–XVII (**68**–**75**)—obtained from a lipophilic fraction of *I. pes-caprae* whole plant ethanol extract [38].

Pescapreins XXI–XXX (**82**–**91**), pentasaccharide resin glycosides, were isolated from 95% ethanol extract of the aerial parts of *I. pes-caprae* [40]. These pescapreins are macrolactones of simonic acid B, partially esterified with different fatty acids. Compounds **82**–**91** were evaluated for their potential to regulate MDR in adriamycin (adriacin doxorubicin, ADR)-resistant human breast cancer cell lines MCF-7/ADR using the standard drug verapamil as a control. Cytotoxicity results of these glycosides revealed that they are non-toxic to MCF-7/ADR. Interestingly, when a 5 μg/mL concentration of these compounds was combined with doxorubicin, the cytotoxicity of doxorubicin was potentiated by 1.5–3.7-fold compared to 21-fold with verapamil. Compounds **82** and **84** are regioisomers of **83** and **85**, respectively. The two pairs of regioisomers (**82** and **83**, and **84** and **85**) showed a large difference in their ability to reverse MDR, demonstrating that a minor structural difference results in a large difference in MDR reversal activity. The IC_50_ value of doxorubicin only and doxorubicin with verapamil is 5.91 and 0.28 μg/mL, respectively, while the IC_50_ values of doxorubicin with each of 82–91 is 1.76, 3.98, 2.00, 3.20, 2.83, 1.58, 3.12, 2.57, 1.82, and 2.60 μg/mL, respectively [40] (Table 8).

**Table 8:** Multidrug resistance efflux inhibiting activity of *I. pes-caprae* natural products.

Compound	Pharmacological Activities	Reference
**58** (Pescaprein II) **59** (Pescaprein III) **61** (Stoloniferin III) **76** (Pescaprein XVIII) **77** (Pescaprein ⅩⅨ) **78** (Pescaprein XX) **79** (Stoloniferin IX) **80** (Stoloniferin X) **81** (Murucoidin VI)	Multidrug-resistance inhibition against *Staphylococcus aureus* SA-1199. A total of 25 μg/mL of each compound potentiated the norfloxacin effect by 4-fold (MIC * from 32 μg/mL to 8 μg/mL)	[39]
**82** (Pescaprein XXI) **83** (Pescaprein XXII) **84** (Pescaprein XXIII) **85** (Pescaprein XXIV) **86** (Pescaprein XXV) **87** (Pescaprein XXVI) **88** (Pescaprein XXVII) **89** (Pescaprein XXVIII) **90** (Pescaprein XXIX) **91** (Pescaprein XXX)	Multidrug-resistance inhibitory effect against MCF-7/ADR ** cells. A total of 5 μg/mL of each compound potentiated the doxorubicin effect by 1.5–3.7-fold, producing IC_50_ *** values of 1.76, 3.98, 2.00, 3.20, 2.83, 1.58, 3.12, 2.57, 1.82, and 2.60 μg/mL for compounds 82–91, respectively	[40]

* MIC: minimum inhibitory concentration; ** MCF-7/ADR: adriamycin (adriacin doxorubicin, ADR)-resistant human breast cancer cell lines; *** IC_50_: half-maximal inhibitory concentration.

### 6.9. Miscellaneous Uses

Apart from the mentioned biological activities, other studies also reported the potential of some metabolites from *I. pes-caprae*. For instance, quercetin 3-*O*-galactoside (**5**), quercetin 3-*O*-glucoside (isoquercetin; **6**), and quercetin (**10**), which were identified from a methanol fraction of aerial parts of *I. pes-caprae*, exhibited angiotensin-converting enzyme (ACE)-inhibitory activity with IC_50_ values of 180, 71, and 151 μM respectively [102]. Moreover, compound **10** showed an excellent immune-regulatory ability by inhibiting TNF-α, interleukin-6 (IL-6), and interleukin-12 (IL-12) production at a 25 μM concentration by 60%, 55%, and 70%, respectively [103].

In other studies, 5-*O*-caffeoylquinic acid (**14**) produced an inhibitory effect on *α*-MSH-induced melanogenesis [47], and caffeic acid (**25**) showed carcinogenic-inhibitory activity [48]. Previous studies have shown **35** to be useful as an anticonvulsant, antiulcer, and antihypertensive agent [57]. (*E*)-Nerolidol (**42**) has anti-biofilm, anti-parasitic, skin-penetration enhancer, and skin-repellent activities [97]. The anti-angiogenic activities using a zebrafish model inhibited intersegmental vessels of embryos treated with 2,4-dihydroxy-6-methoxyacetophenone (**93**) with an IC_50_ value of 0.083 μM [100].

Glucosidase inhibitors are promising therapeutic potential in treating diabetes, human immunodeficiency virus (HIV) infection, metastatic cancer, and lysosomal storage diseases [104]. Studies conducted on rat lysosomes suggested the presence of these inhibitors in *I. pes-caprae*. Contrary to Meira et al., which reported the presence of calystegine B1, B2 (**1**), B3, and C1 in *I. pes-caprae* [89], only the evidence of calystegine B2 (**1**) from *I. pes-caprae* exists from a reference found [29]. The polyhydroxylated alkaloid **1** was previously isolated from *Ipomoea carnea* and evaluated for the inhibition of rat epididymis glycosidases. From the result obtained, compound **1** proved potent by inhibiting lysosomal *β*-glucosidase with an IC_50_ value of 0.75 μM, representing the best inhibitory activity among the tested calystegines, including B1, B3, and C1 [105]. Resin glycosides play important roles in the purgative properties of some Convolvulaceae species [106] (Table 9).

**Table 9:** Miscellaneous uses of *I. pes-caprae* natural products.

Compound	Pharmacological Activities	Reference
**1** (Calystegine B2)	Potent inhibitory activity toward rat lysosomal *β*-glucosidase (IC_50_ * = 0.75 µM)	[105]
**5**	Inhibitory activity against ACE ** (IC_50_ = 180 μM)	[102]
**6** (Isoquercetin)	Inhibitory activity against ACE (IC_50_ = 71 μM)	
**10** (Quercetin)	Inhibitory activity against ACE (IC_50_ = 151 μM) 25 μM concentration inhibited TNF-α ***, IL-6 ****, and IL-12 ***** production at 60%, 55%, and 70%, respectively	[102,103]
**93**	Anti-angiogenic activities (IC_50_ = 0.083 μM)	[100]

* IC_50_: half-maximal inhibitory concentration; ** ACE: angiotensin-converting enzyme; *** TNF-α: tumor necrosis factor-α; **** IL-6: interleukin 6; ***** IL-12: interleukin 12.

## 7. Materials and Methods

The information on the botanical description, traditional uses, and bioactivities of the secondary metabolites from *Ipomoea pes-caprae* were searched through a combination of databases, which include Google Scholar, PubMed, ScienceDirect, DOAJ, and SpringerLink. The data was updated until December 2021 using ‘biological activities of *Ipomoea pes-caprae*,’ ‘*Ipomoea pes-caprae* natural products,’ ‘*I. pes-caprae* traditional uses,’ ‘invasive and introduced *I. pes-caprae*’ and ‘*I. pes-caprae* botany’ as key search phrases. Publications were excluded from this review that did not or inadequately provide information on isolated and identified compounds from *I. pes-caprae*, geographical distribution, botanical description, traditional uses, and bioactivities of the extract. The study also excluded primary metabolites with no known biological activity from *I. pes-caprae*. Some of the components from the gas chromatography–mass spectrometry (GC-MS) analysis of *I. pes-caprae* leaves essential oil were included as secondary metabolites. The qantitative bioactivity information was selected to be included in the Tables.

## 8. Conclusions

*Ipomoea pes-caprae* is a widely distributed medicinal halophyte in tropical and subtropical regions, where it serves many uses in traditional medicine. *I. pes-caprae* is one of the most widely distributed beach plants across the world. The whole plant can be collected along coastal beaches, seashores, and sandy beaches, followed by proper identification and deposition of collected species in an herbarium. In some cases, the plant can be purchased and identified by a taxonomist or through comparison with an authentic sample collected in the herbarium. The plant samples of *I. pes-caprae* discussed in this review included collections from India, Brazil, Mexico, Mauritius, Thailand, Japan, and China, where the plant is used for food and therapeutic purposes. The biological activities of *I. pes-caprae* extract include antioxidant, anti-inflammatory, antinociceptive, antimicrobial, antispasmodic, anticancer, antitumor, antiproliferative, and multidrug-resistance inhibitory activities. The intrinsic biological potential and medicinal use of *I. pes-caprae* can be associated with many phytochemicals, such as alkaloids, norisoprenoids, phenols, terpenoids, steroids, and glycosides, contained in the plant.

For treating gastrointestinal-related disorders and symptoms, such as dysentery, ulcer, abdominal pain, and cramps, the leaves, seeds, and roots of *I. pes-caprae* are used. The use of this plant for this purpose can be attributed to the antispasmodic and antiulcerative effect of compounds, such as isochlorogenic acid A (**19**; yield 0.279 mg/g), *E*-phytol (**32**; yield 0.006 mg/g), *β*-damascenone (**50**; yield 0.0005 mg/g), and xanthoxyline (**92**; yield 0.003 mg/g). Either in isolation or synergy, the amount of these compounds contained in infusion or decoction of *I. pes-caprae* is enough to elicit effects capable of soothing abdominal pains and cramps and treating ulcers as used in traditional medicine. Furthermore, resin glycosides from the family Convolvulaceae are known for their purgative abilities, thus confirming the medicinal values of *I. pes-caprae* in traditional medicine for treating digestive disorders.

For treating skin diseases and associated pain and inflammations, such as dermatitis, boil, sores, earache, and stings from jellyfish and stony fish, the leaves and stem sap of *I. pes-caprae* are used. This review has shown that *I. pes-caprae* contains a host of compounds with antinociceptive and anti-inflammatory activities that support its use for the treatment of the toxic effects of jellyfish venoms, dermatitis, arthritis, rheumatism, and to manage internal and external pains in traditional medicine. These compounds include actinidols la (**2**) and lb (**3**), isoquercetin (**6**; yield 0.026 mg/g), quercetin (**10**; yield 0.006 mg/g), (-) mellein (**11**; yield 0.001 mg/g), eugenol (**15**; yield 0.005 mg/g), and 4-vinylguaiacol (**16**; yield 0.0005 mg/g), 2-hydroxy-4,4,7-trimethyl-1(4*H*)-naphthalenone (**31**; yield 0.002 mg/g), *E*-phytol (**32**), *α*-amyrin (**35**; yield 0.004 mg/g), *β*-amyrin (**36**; yield 0.002 mg/g), *α*-amyrin acetate (**37**; yield 0.003 mg/g), *β*-amyrin acetate (**38**; yield 0.003 mg/g), betulinic acid (**39**; yield 0.0006 mg/g), and glochidone (**40**; yield 0.009 mg/g). The use of *I. pes-caprae* to treat pains and inflammations can be associated with the effects of these identified compounds. Additionally, some bacteria, such as *Staphylococcus aureus*, are responsible for certain skin diseases and inflammations. Compounds, such as (-) mellein (**11**), chlorogenic acid (**14**), caffeic acid (**25**), *β*-caryophyllene (**46**), and *β*-sitosterol (**55**; yield 0.008 mg/g), are important antimicrobial agents from this plant. As such, a combination of the antimicrobial and anti-inflammatory activities of these compounds clearly shows why this plant is used to treat skin diseases and inflammations. Over 30 resin glycosides were reported from *I. pes-caprae* in this review, with stoloniferin IX (**79**; yield 0.05 mg/g) and pescaprein XX (**78**; yield 0.004 mg/g) having the best yields among others. These glycosides (**78** and **79**) showed effectiveness by inhibiting multidrug resistance and potentiating the action of norfloxacin against the NorA overexpressing bacteria strains. Therefore, these glycosides can potentiate the antibacterial effect of other compounds, thus making the activity more pronounced, providing some support for the use of the plant for treating many bacteria-related diseases.

Another use of *I. pes-caprae* in traditional medicine is to alleviate strain, fatigue, and physical weakness. For this purpose, the leaves and the whole plant have been used as infusions or decoctions. Excess free radicals are involved in the pathogenesis of different diseases and conditions, such as strain, fatigue, and physical weakness. Compounds, namely, 5,7-dihydroxy-4-phenyl-2*H*-chromen-2-one (**4**), isoquercetin (**6**) quercetin (**10**), chlorogenic acid (**14**), isochlorogenic acid B (**21**; yield 0.059 mg/g), isochlorogenic acid C (**23**; yield 0.168 mg/g), caffeic acid (**25**), *β*-amyrin (**36**), *β*-caryophyllene (**46**), stigmasterol (**54**; yield 0.002 mg/g), and *β*-sitosterol (**55**), are all known to possess antioxidant activities. These *I. pes-caprae* compounds act by scavenging free radicals, contributing wholly or partly to mitigating strain, fatigue, and physical weakness.

The use of *I. pes-caprae* for treating hypertension and kidney ailments has also been reported in the literature. Many anti-hypertensive drugs have good antioxidant properties, useful for improving vascular function and reducing blood pressure in animal models and humans. This study has shown that *I. pes-caprae* houses many compounds with proven antioxidant properties, such as **4**, **6**, **10**, **14**, **21**, **23**, **25**, **36**, **46**, **54**, and **55**. The amount of these compounds in *I. pes-caprae* is enough to validate the effectiveness of this plant to treat hypertension traditionally. Furthermore, antibiotics serve as a therapy for kidney diseases, such as those caused by *E. coli*. Thus, compounds, such as **14**, **25**, **32**, **46**, **53**, and **55**, with good antimicrobial profiles might play important roles in the treatment of kidney ailments with the use of *I. pes-caprae*.

*Ipomoea pes-caprae* is a key component of Ayurvedic medicine, especially for the treatment of diabetes. The presence of the potent glucosidase inhibitor—calystegine B2 (**1**)—pexplains the therapeutic potential of this plant as used in traditional medicine for treating diabetes. Additionally, the antioxidant compounds from *I. pes-caprae* are also important for managing diabetes. *I. pes-caprae* is also used to treat hemorrhoids, which often present symptoms similar to digestive disorders accompanied by pain and discomfort. The identified compounds with digestive order and pain-soothing effects support using *I. pes-caprae* locally for this purpose.

The evaluation of the biological activity of *I. pes-caprae* was mostly conducted on the leaves and stems, while a dearth of information exists on other plant parts. Since different plant parts can show different biological activities, more studies should be conducted on other parts of *I. pes-caprae*, such as its flowers, fruits, seeds, or roots. Moreover, even though many studies have been conducted on *I. pes-caprae*, these studies are often limited to Asia, with a paucity of scientific evidence on the bioactivity of this plant from other regions, such as Africa, America, and Australia, where they are known to occur.

Although *I. pes-caprae* is considered a tropical plant growing around coastal areas, there are now pieces of evidence and report of the occurrence of *I. pes-caprae* as invasive species in areas outside the tropics, such as Spain, South Africa, Anguilla, and Marshall Island, spreading and gradually establishing itself by out-competing the native species. Large populations of this plant have been found growing in inland habitats, even though they are originally associated with coastal areas. The establishment of invasive species in these new habitats could be because the invasive species respond to climate change better than the native species. Therefore, the influence of climate change, most importantly global warming, cannot be overemphasized as having a key effect on the dispersal and establishment of *I. pes-caprae* into these new climatic regions.

This review provides insights that may facilitate the future study of the phytochemistry of this plant. *I. pes-caprae* is native to saline habitats, where the soil mixture contains organic nutrient material and inorganic nutrient salts flowing from the land and ocean, and are thereby expected to possess abundant biologically active secondary metabolites, such as vitamins, terpenoids (carotenoids and essential oils), and phenolics, due to these extreme conditions. Further evaluation of this halophyte for biological assays not suggested in traditional medicine is required. Research on *I. pes-caprae* has been restricted to the stems and leaves. Other *I. pes-caprae* parts, such as the flowers and fruits, should be further explored for more bioactive secondary metabolites. More studies should be conducted to evaluate the structure–activity relationship and mechanism of action for some identified compounds obtained from this species. This study has shown that *I. pes-caprae* contains important bioactive secondary metabolites. Finally, since climatic differences and habitat can influence the biosynthesis of secondary metabolites, this study strongly suggests that the further evaluation of the phytochemistry of both the invasive and inland species of *I. pes-caprae* be conducted, to make possible the comparison of their respective phytochemicals with the reported studies.

## Data Availability

No new data were created or analyzed in this study. Data sharing is not applicable to this article.

## References

[1] Devall M.S. (1992). The Biological Flora of Coastal Dunes and Wetlands. 2. *Ipomoea pes-caprae* (L.) Roth. J. Coast. Res..

[2] Manigaunha A., Ganesh N., Kharya M.D. (2010). Morning Glory: A New Thirst In-Search of De-Novo Therapeutic Approach. Int. J. Phytomed..

[3] Pereda-Miranda R., Escalante-Sánchez E., Escobedo-Martínez C. (2005). Characterization of Lipophilic Pentasaccharides from Beach Morning Glory (*Ipomoea pes-caprae*). J. Nat. Prod..

[4] Emendörfer F., Emendörfer F., Bellato F., Noldin V.F., Niero R., Cechinel-Filho V., Cardozo A.M. (2005). Evaluation of the Relaxant Action of Some Brazilian Medicinal Plants in Isolated Guinea-Pig Ileum and Rat Duodenum. J. Pharm. Pharmaceut. Sci..

[5] Pothula V.V.S., Kanikaram S. (2015). In Vitro Antiplasmodial Efficacy of Mangrove Plant, *Ipomoea pes-caprae* against *Plasmodium falciparum* (3D7 Strain). Asian Pac. J. Trop. Dis..

[6] Brown S.H., Frank M.S. (2020). Railroad Vine (*Ipomoea pes-caprae*): Identification and Uses. Edis.

[7] Nilam R., Jyoti P., Sumitra C. (2018). Pharmacognostic and Phytochemical Studies of *Ipomoea pes-caprae*, an Halophyte from Gujarat. J. Pharmacogn. Phytochem..

[8] Miryeganeh M., Takayama K., Tateishi Y., Kajita T. (2014). Long-Distance Dispersal by Sea-Drifted Seeds Has Maintained the Global Distribution of *Ipomoea pes-caprae* Subsp. *Brasiliensis* (Convolvulaceae). PLoS ONE.

[9] Ouyang P.Y., Liu N., Zhang W.W., Wang J., Jian S.G. (2011). Biological and Ecophysiological Characteristics of a Beach Plant *Ipomoea pes-caprae*. J. Hunan Univ. Sci. Technol. (Nat. Sci. Ed.).

[10] Okui T., Nohara S., Furukawa A. (2003). The Role of Adventitious Roots in Supplying Water to *Ipomoea pes-caprae*. Tropics.

[11] Kamakura M., Furukawa A. (2009). Compensatory Function for Water Transport by Adventitious Roots of *Ipomoea pes-caprae*. J. Plant Res..

[12] Zhang M., Zhang H., Zheng J.X., Mo H., Xia K.F., Jian S.G. (2018). Functional Identification of Salt-Stress-Related Genes Using the Fox Hunting System from *Ipomoea pes-caprae*. Int. J. Mol. Sci..

[13] Suarez N. (2011). Comparative Leaf Anatomy and Pressure-Volume Analysis in Plants of *Ipomoea pes-caprae* Experimenting Saline and/or Drought Stress. Int. J. Bot..

[14] *Ipomoea pes-caprae* (L.) R. Br. https://www.gbif.org/species/7999386.

[15] Devall M.S., Thien L.B. (2005). Inland Occurrence of the Strand Plant *Ipomoea pes-caprae* (Convolvulaceae) around Lake Nicaragua. Southwest. Nat..

[16] St. John H. (1970). Classification and Distribution of the *Ipomoea pes-caprae* Group (Convolvulaceae). Bot. Jahrebucher.

[17] Ridley H.N. (1930). The Dispersal and Plants throughout the World.

[18] Pongprayoon U., Bohlin L., Sandberg F. (1989). Inhibitory Effect of Extract of *Ipomoea pes-caprae* on Guinea-Pig Ileal Smooth Muscle. Acta Pharm. Nord..

[19] Chan E.W.C., Baba S., Chan H.T., Kainuma M., Tangah J. (2016). Medicinal Plants of Sandy Shores: A Short Review on *Vitex trifolia* L. and *Ipomoea pes-caprae* (L.) R. Br. Indian J. Nat. Prod. Resour..

[20] Marie D.E.P., Dejan B., Quetin-Leclercq J. (2007). GC-MS Analysis of the Leaf Essential Oil of *Ipomoea pes-caprae*, a Traditional Herbal Medicine in Mauritius. Nat. Prod. Commun..

[21] Iwu M.M., Anyanwu B.N. (1982). Phytotherapeutic Profile of Nigerian Herbs. I: Anti-Inflammatory and Anti-Arthritic Agents. J. Ethnopharmacol..

[22] Teerakulkittipong N., Phosri S., Chetkhetkran M. (2020). Effect of Extraction Methods on Yield, Total Phenolic Content and Antioxidant Activity of *Ipomoea pes-caprae* (L.) R. Br. Leaves. Proc. RSU Int. Res. Conf..

[23] Zhao K., Feng L. (2001). Resource of Halophytic Vegetation in China.

[24] Krogh R., Kroth R., Berti C., Madeira A.O., Souza M.M., Cechinel-Filho V., Delle-Monache F., Yunes R.A. (1999). Isolation and Identification of Compounds with Antinociceptive Action from *Ipomoea pes-caprae* (L.) R. Br. Pharmazie.

[25] Salguero C.P. (2003). A Compendium of Traditional Thai Herbal Medicine. A Thai Herbal: Traditional Recipes for Health and Harmony.

[26] Lorenzi H., Matos F.J.A. (2002). Plantas Medicinais No Brasil: Nativas e Exóticas. Instituto Plantarum Nova Odessa.

[27] Vivek P., Jayakumari D., Jayasree P. (2016). Hypoglycaemic Effect of Vriddhadaru [*Argyreia nervosa* (Burm. F.) Boj.] in Alloxan Induced Diabetic Rabbits. Int. J. Adv. Ayurveda Yoga Unani Siddha Homeopath..

[28] Manigauha A., Kharya M.D., Ganesh N. (2015). In Vivo Antitumor Potential of *Ipomoea pes-caprae* on Melanoma Cancer. Pharmacogn. Mag..

[29] Schimming T., Jenett-Siems K., Mann P., Tofern-Reblin B., Milson J., Johnson R.W., Deroin T., Austin D.F., Eich E. (2005). Calystegines as Chemotaxonomic Markers in the Convolvulaceae. Phytochemistry.

[30] Asano N., Yokoyama K., Sakurai M., Ikeda K., Kizu H., Kato A., Arisawa M., Höke D., Dräger B., Watson A.A. (2001). Dihydroxynortropane Alkaloids from Calystegine-Producing Plants. Phytochemistry.

[31] Pongprayoon U., Bohlin L., Baeckstrom P., Jacobson U., Lindstrom M. (1992). Inhibition of Ethyl Phenylpropiolate-Induced Rat Ear Oedema by Compounds Isolated from *Ipomoea pes-caprae* (L.) R. Br. Phytother. Res..

[32] Alagesan V., Ramalingam S., Kim M., Venugopal S. (2019). Antioxidant Activity Guided Isolation of a Coumarin Compound from *Ipomoea pes-caprae* (Convolvulaceae) Leaves Acetone Extract and Its Biological and Molecular Docking Studies. Eur. J. Integr. Med..

[33] Gonçalves F.M.B., Ramos A.C., da Silva Mathias M., de Souza Sales Q., Ramos C.C., Antunes F., de Oliveira R.R. (2020). Phytochemical Analysis and Hypotensive Activity of *Ipomoea pes-caprae* on Blood Pressure of Normotensive Rats. Rodriguesia.

[34] Pongprayoon U., Baeckström P., Jacobsson U., Lindström M., Bohlin L. (1991). Compounds Inhibiting Prostaglandin Synthesis Isolated from *Ipomoea pes-caprae*. Planta Med..

[35] Teramachi F., Koyano T., Kowithayakorn T., Hayashi M., Komiyama K., Ishibashi M. (2005). Collagenase Inhibitory Quinic Acid Esters from *Ipomoea pes-caprae*. J. Nat. Prod..

[36] Pongprayoon U., Baeckstrom P., Jacobsson U., Lindstrom M., Bohlin L. (1992). Antispasmodic Activity of β-Damascenone and E-Phytol Isolated from *Ipomoea pes-caprae*. Planta Med..

[37] Escobedo-Martínez C., Pereda-Miranda R. (2007). Resin Glycosides from *Ipomoea pes-caprae*. J. Nat. Prod..

[38] Tao H., Hao X., Liu J., Ding J., Fang Y., Gu Q., Zhu W. (2008). Resin Glycoside Constituents of *Ipomoea pes-caprae* (Beach Morning Glory). J. Nat. Prod..

[39] Escobedo-Martínez C., Cruz-Morales S., Fragoso-Serrano M., Mukhlesur Rahman M., Gibbons S., Pereda-Miranda R. (2010). Characterization of a Xylose Containing Oligosaccharide, an Inhibitor of Multidrug Resistance in *Staphylococcus aureus*, from *Ipomoea pes-aprae*. Phytochemistry.

[40] Yu B.W., Luo J.G., Wang J.S., Zhang D.M., Yu S.S., Kong L.Y. (2011). Pentasaccharide Resin Glycosides from *Ipomoea pes-caprae*. J. Nat. Prod..

[41] Qasim M., Abideen Z., Adnan M.Y., Gulzar S., Gul B., Rasheed M., Khan M.A. (2017). Antioxidant Properties, Phenolic Composition, Bioactive Compounds and Nutritive Value of Medicinal Halophytes Commonly Used as Herbal Teas. S. Afr. J. Bot..

[42] Yang D., Wang T., Long M., Li P. (2020). Quercetin: Its Main Pharmacological Activity and Potential Application in Clinical Medicine. Oxid. Med. Cell. Longev..

[43] Majewska M., Skrzycki M., Podsiad M., Czeczot H. (2011). Evaluation of Antioxidant Potential of Flavonoids: An In Vitro Study. Acta Pol. Pharm.-Drug Res..

[44] Sunil C., Irudayaraj S.S., Duraipandiyan V., Al-Dhabi N.A., Agastian P., Ignacimuthu S. (2014). Antioxidant and Free Radical Scavenging Effects of β-Amyrin Isolated from *S. cochinchinensis* Moore. Leaves. Ind. Crops Prod..

[45] Ayaz M., Junaid M., Ullah F., Subhan F., Sadiq A., Ali G., Ovais M., Shahid M., Ahmad A., Wadood A. (2017). Anti-Alzheimer’s Studies on β-Sitosterol Isolated from *Polygonum hydropiper* L. Front. Pharmacol..

[46] Pedersen J.Z., Oliveira C., Incerpi S., Kumar V., Fiore A.M., de Vito P., Prasad A.K., Malhotra S., Parmar V.S., Saso L. (2010). Antioxidant Activity of 4-Methylcoumarins. J. Pharm. Pharmacol..

[47] Kim H.H., Kim J.K., Kim J., Jung S.H., Lee K. (2020). Characterization of Caffeoylquinic Acids from Lepisorus Thunbergianus and Their Melanogenesis Inhibitory Activity. ACS Omega.

[48] Magnani C., Isaac V.L.B., Correa M.A., Salgado H.R.N. (2014). Caffeic Acid: A Review of Its Potential Use in Medications and Cosmetics. Anal. Methods.

[49] Gao Y., Fang L., Wang X., Lan R., Wang M., Du G., Guan W., Liu J., Brennan M., Guo H. (2019). Antioxidant Activity Evaluation of Dietary Flavonoid Hyperoside Using *Saccharomyces cerevisiae* as a Model. Molecules.

[50] Razavi S.M., Zahri S., Zarrini G., Nazemiyeh H., Mohammadi S. (2009). Biological Activity of Quercetin-3-O-Glucoside, a Known Plant Flavonoid. Russ. J. Bioorg. Chem..

[51] Chiang Y.M., Chuang D.Y., Wang S.Y., Kuo Y.H., Tsai P.W., Shyur L.F. (2004). Metabolite Profiling and Chemopreventive Bioactivity of Plant Extracts from *Bidens pilosa*. J. Ethnopharmacol..

[52] Rivero-cruz J.F., Granados-pineda J., Pedraza-chaverri J., Rivero-cruz B.E. (2020). Phytochemical Constituents, Antioxidant, Cytotoxic, and Antimicrobial Activities of the Ethanolic Extract of Mexican Brown *Propolis*. Antioxidants.

[53] Randjelović P., Veljković S., Stojiljković N., Sokolović D., Ilić I., Laketić D., Randjelović D., Randjelović N. (2015). The Beneficial Biological Properties of Salicylic Acid. Acta Fac. Med. Naissensis.

[54] Li Z.H., Cai M., Liu Y.S., Sun P.L., Luo S.L. (2019). Antibacterial Activity and Mechanisms of Essential Oil from *Citrus medica* L. Var. Sarcodactylis. Molecules.

[55] Dahham S.S., Tabana Y.M., Iqbal M.A., Ahamed M.B.K., Ezzat M.O., Majid A.S.A., Majid A.M.S.A. (2015). The Anticancer, Antioxidant and Antimicrobial Properties of the Sesquiterpene β-Caryophyllene from the Essential Oil of *Aquilaria crassna*. Molecules.

[56] Türkez H., Çelik K., Toğar B. (2014). Effects of Copaene, a Tricyclic Sesquiterpene, on Human Lymphocytes Cells In Vitro. Cytotechnology.

[57] Khaleel C., Tabanca N., Buchbauer G. (2018). α-Terpineol, a Natural Monoterpene: A Review of Its Biological Properties. Open Chem..

[58] Tepe B., Akpulat H.A., Sokmen M. (2011). Evaluation of the Chemical Composition and Antioxidant Activity of the Essential Oils of *Peucedanum longifolium* (Waldst. & Kit.) and *P. palimbioides* (Boiss.). Rec. Nat. Prod..

[59] Azab A., Nassar A., Azab A.N. (2016). Anti-Inflammatory Activity of Natural Products. Molecules.

[60] Maione F., Russo R., Khan H., Mascolo N. (2016). Medicinal Plants with Anti-Inflammatory Activities. Nat. Prod. Res..

[61] Pongprayoon U., Bohlin L., Soonthornsaratune P., Wasuwat S. (1991). Antiinflammatory Activity of *Ipomoea pes-caprae* (L.) R. Br. Phytother. Res..

[62] Davis D.L., Stevens K.L., Jurd L. (1976). Chemistry of Tobacco Constituents. Oxidation of α-Ionone and the Acid-Catalyzed Rearrangement of 5-Keto-α-Ionone. J. Agric. Food Chem..

[63] Magalhães C.B., Riva D.R., Depaula L.J., Brando-Lima A., Koatz V.L.G., Leal-Cardoso J.H., Zin W.A., Faffe D.S. (2010). In Vivo Anti-Inflammatory Action of Eugenol on Lipopolysaccharide-Induced Lung Injury. J. Appl. Physiol..

[64] Li Y., Yao J., Han C., Yang J., Chaudhry M.T., Wang S., Liu H., Yin Y. (2016). Quercetin, Inflammation and Immunity. Nutrients.

[65] Ruangnoo S., Jaiaree N., Makchuchit S., Panthong S., Thongdeeying P., Itharat A. (2012). An In Vitro Inhibitory Effect on RAW 264.7 Cells by Antiinflammatory Compounds from *Smilax corbularia* Kunth. Asian Pac. J. Allergy Immunol..

[66] Chavan M.J., Wakte P.S., Shinde D.B. (2010). Analgesic and Anti-Inflammatory Activity of Caryophyllene Oxide from *Annona squamosa* L. Bark. Phytomedicine.

[67] Ghavam M., Manca M.L., Manconi M., Bacchetta G. (2020). Chemical Composition and Antimicrobial Activity of Essential Oils Obtained from Leaves and Flowers of *Salvia hydrangea* DC. Ex Benth. Sci. Rep..

[68] Gupta M.B., Bhalla T.N., Tangri K.K., Bhargava K.P. (1971). Biochemical Study of the Anti-Inflammatory Activity of α and β-Amyrin Acetate. Biochem. Pharmacol..

[69] Safayhi H., Sailer E.R. (1997). Anti-Inflammatory Actions of Pentacyclic Triterpenes. Planta Med..

[70] Velázquez-González C., Cariño-Cortés R., Gayosso de Lucio J.A., Ortiz M.I., de la O Arciniega M., Altamirano-Báez D.A., Ángeles L.J., Bautista-Ávila M. (2014). Antinociceptive and Anti-Inflammatory Activities of *Geranium bellum* and Its Isolated Compounds. BMC Complement. Altern. Med..

[71] Maria De Souza M., Madeira A., Berti C., Krogh R., Yunes R.A., Cechinel-Filho V. (2000). Antinociceptive Properties of the Methanolic Extract Obtained from *Ipomoea pes-caprae* (L.) R. Br. J. Ethnopharmacol..

[72] Otuki M.F., Ferreira J., Lima F., Meyre-Silva C., Malheiros Â., Muller L.A., Cani G.S., Santos A.R.S., Yunes R.A., Calixto J.B. (2005). Antinociceptive Properties of Mixture of α-Amyrin and β-Amyrin Triterpenes: Evidence for Participation of Protein Kinase C and Protein Kinase A Pathways. J. Pharmacol. Exp. Ther..

[73] Ghaffari Moghaddam M., Ahmad F.B.H., Samzadeh-Kermani A. (2012). Biological Activity of Betulinic Acid: A Review. Pharmacol. Amp. Pharm..

[74] Quintans-Júnior L., Moreira J.C.F., Pasquali M.A.B., Rabie S.M.S., Pires A.S., Schröder R., Rabelo T.K., Santos J.P.A., Lima P.S.S., Cavalcanti S.C.H. (2013). Antinociceptive Activity and Redox Profile of the Monoterpenes (+)-Camphene, p -Cymene, and Geranyl Acetate in Experimental Models. ISRN Toxicol..

[75] Nagababu P., Umamaheswara Rao V. (2015). Pharmacological Potential of *Ipomea pes-caprae* (L.) R. Br. Whole Plant Extracts. Pelagia Res. Libr. Pharm. Sin..

[76] Ghaneian M.T., Ehrampoush M.H., Jebali A., Hekmatimoghaddam S., Mahmoudi M. (2015). Antimicrobial Activity, Toxicity and Stability of Phytol as a Novel Surface Disinfectant. Environ. Health Eng. Manag. J..

[77] Sen A., Dhavan P., Shukla K.K., Singh S., Tejovathi G. (2012). Analysis of IR, NMR and Antimicrobial Activity of β-Sitosterol Isolated from *Momordica charantia*. Sci. Secur. J. Biotech..

[78] Lou Z., Wang H., Zhu S., Ma C., Wang Z. (2011). Antibacterial Activity and Mechanism of Action of Chlorogenic Acid. J. Food Sci..

[79] Kȩpa M., Miklasińska-Majdanik M., Wojtyczka R.D., Idzik D., Korzeniowski K., Smoleń-Dzirba J., Wasik T.J. (2018). Antimicrobial Potential of Caffeic Acid against *Staphylococcus aureus* Clinical Strains. BioMed Res. Int..

[80] Toyama D.O., Ferreira M.J.P., Romoff P., Fávero O.A., Gaeta H.H., Toyama M.H. (2014). Effect of Chlorogenic Acid (5-Caffeoylquinic Acid) Isolated from *Baccharis oxyodonta* on the Structure and Pharmacological Activities of Secretory Phospholipase A2 from *Crotalus durissus terrificus*. BioMed Res. Int..

[81] Zhang X., Huang H., Yang T., Ye Y., Shan J., Yin Z., Luo L. (2010). Chlorogenic Acid Protects Mice against Lipopolysaccharide-Induced Acute Lung Injury. Injury.

[82] Cheng S.S., Wu C.L., Chang H.T., Kao Y.T., Chang S.T. (2004). Antitermitic and Antifungal Activities of Essential Oil of *Calocedrus formosana* Leaf and Its Composition. J. Chem. Ecol..

[83] Garcia M.C.F., Soares D.C., Santana R.C., Saraiva E.M., Siani A.C., Ramos M.F.S., Danelli M.D.G.M., Souto-Padron T.C., Pinto-Da-Silva L.H. (2018). The In Vitro Antileishmanial Activity of Essential Oil from *Aloysia gratissima* and Guaiol, Its Major Sesquiterpene against Leishmania Amazonensis. Parasitology.

[84] Mbunde M.V.N., Innocent E., Mabiki F., Andersson P.G. (2021). In Vitro Study for Antifungal Compounds from *Parinari curatellifolia* (Chrysobalanaceae) and *Terminalia sericea* (Combretaceae). Int. J. Biol. Chem. Sci..

[85] Sieniawska E., Swatko-Ossor M., Sawicki R., Skalicka-Woźniak K., Ginalska G. (2017). Natural Terpenes Influence the Activity of Antibiotics against Isolated *Mycobacterium tuberculosis*. Med. Princ. Pract..

[86] Pinheiro T.R., Yunes R.A., López S.N., Santecchia C.B., Zacchino S.A.S., Cechinel Filho V. (1999). In Vitro Antifungal Evaluation and Studies on the Mode of Action of Xanthoxyline Derivatives. Arzneim.-Forsch./Drug Res..

[87] Abdelwahab A.B., Salwinski A., Simonsen H.T. (2021). Collagenase and Tyrosinase Inhibitory Effect of Isolated Constituents from the Moss *Polytrichum formosum*. Plants.

[88] Zoofishan Z., Kúsz N., Csorba A., Tóth G., Hajagos-Tóth J., Kothencz A., Gáspár R., Hunyadi A. (2019). Antispasmodic Activity of Prenylated Phenolic Compounds from the Root Bark of *Morus nigra*. Molecules.

[89] Meira M., Pereira Da Silva E., David J.M., David J.P. (2012). Review of the Genus *Ipomoea*: Traditional Uses, Chemistry and Biological Activities. Rev. Bras. Farmacogn. Braz. J. Pharmacogn..

[90] Cechinel Filho V., Gomes Miguel O., José Nunes R., Batista Calixto J., Augusto Yunes R. (1995). Antispasmodic Activity of Xanthoxyline Derivatives: Structure-Activity Relationships. J. Pharm. Sci..

[91] Lichota A., Gwozdzinski K. (2018). Anticancer Activity of Natural Compounds from Plant and Marine Environment. Int. J. Mol. Sci..

[92] Kang T.B., Liang N.C. (1997). Studies on the Inhibitory Effects of Quercetin on the HL-60 Leukemia Cells. Biochem. Pharmacol..

[93] Chou C.C., Yang J.S., Lu H.F., Ip S.W., Lo C., Wu C.C., Lin J.P., Tang N.Y., Chung J.G., Chou M.J. (2010). Quercetin-Mediated Cell Cycle Arrest and Apoptosis Involving Activation of a Caspase Cascade through the Mitochondrial Pathway in Human Breast Cancer MCF-7 Cells. Arch. Pharm. Res..

[94] Li K., Yuan D., Yan R., Meng L., Zhang Y., Zhu K. (2018). Stigmasterol Exhibits Potent Antitumor Effects in Human Gastric Cancer Cells Mediated via Inhibition of Cell Migration, Cell Cycle Arrest, Mitochondrial Mediated Apoptosis and Inhibition of JAK/STAT Signalling Pathway. J. BUON.

[95] Dinku W., Isaksson J., Rylandsholm F.G., Bouř P., Brichtová E., Choi S.U., Lee S.H., Jung Y.S., No Z.S., Svendsen J.S.M. (2020). Anti-Proliferative Activity of a Novel Tricyclic Triterpenoid Acid from *Commiphora africana* Resin against Four Human Cancer Cell Lines. Appl. Biol. Chem..

[96] Vo T.K., Ta Q.T.H., Chu Q.T., Nguyen T.T., Vo V.G. (2020). Anti-Hepatocellular-Cancer Activity Exerted by β-Sitosterol and β-Sitosterol-Glucoside from *Indigofera zollingeriana* Miq. Molecules.

[97] Chan W.K., Tan L.T.H., Chan K.G., Lee L.H., Goh B.H. (2016). Nerolidol: A Sesquiterpene Alcohol with Multi-Faceted Pharmacological and Biological Activities. Molecules.

[98] Yang C., Chen H., Chen H., Zhong B., Luo X., Chun J. (2017). Antioxidant and Anticancer Activities of Essential Oil from Gannan Navel Orange Peel. Molecules.

[99] Zhang X., Hu J., Chen Y. (2016). Betulinic Acid and the Pharmacological Effects of Tumor Suppression (Review). Mol. Med. Rep..

[100] Yang D., He Q., Yang Y., Liu K., Li X. (2014). Chemical Constituents of *Euphorbia tibetica* and Their Biological Activities. Chin. J. Nat. Med..

[101] Lira-Ricárdez J., Pereda-Miranda R. (2020). Reversal of Multidrug Resistance by Amphiphilic Morning Glory Resin Glycosides in Bacterial Pathogens and Human Cancer Cells. Phytochem. Rev..

[102] Balasuriya N., Rupasinghe H.P.V. (2012). Antihypertensive Properties of Flavonoid-Rich Apple Peel Extract. Food Chem..

[103] Ho S.T., Tung Y.T., Wu Y.J., Lin C.C., Wu J.H. (2015). Immune-Regulatory Activity of Methanolic Extract of *Acacia confusa* Heartwood and Melanoxetin Isolated from the Extract. Holzforschung.

[104] Borges de Melo E., da Silveira Gomes A., Carvalho I. (2006). α- and β-Glucosidase Inhibitors: Chemical Structure and Biological Activity. Tetrahedron.

[105] Haraguchi M., Gorniak S.L., Ikeda K., Minami Y., Kato A., Watson A.A., Nash R.J., Molyneux R.J., Asano N. (2003). Alkaloidal Components in the Poisonous Plant, *Ipomoea carnea* (Convolvulaceae). J. Agric. Food Chem..

[106] Pereda-Miranda R., Bah M. (2003). Biodynamic Constituents in the Mexican Morning Glories: Purgative Remedies Transcending Boundaries. Curr. Top. Med. Chem..

